# Bacteria extracellular vesicle as nanopharmaceuticals for versatile biomedical potential

**DOI:** 10.1186/s40580-024-00434-5

**Published:** 2024-07-11

**Authors:** Ming Yao Ho, Songhan Liu, Bengang Xing

**Affiliations:** School of Chemistry, Chemical Engineering and Biotechnology, Nanyang Technological University, 21 Nanyang Link, Singapore, S637371 Singapore

**Keywords:** Bacterial extracellular vesicle, Nanomedicine, Drug delivery, Vaccination, Nanotechnology, Immunotherapy

## Abstract

Bacteria extracellular vesicles (BEVs), characterized as the lipid bilayer membrane-surrounded nanoparticles filled with molecular cargo from parent cells, play fundamental roles in the bacteria growth and pathogenesis, as well as facilitating essential interaction between bacteria and host systems. Notably, benefiting from their unique biological functions, BEVs hold great promise as novel nanopharmaceuticals for diverse biomedical potential, attracting significant interest from both industry and academia. Typically, BEVs are evaluated as promising drug delivery platforms, on account of their intrinsic cell-targeting capability, ease of versatile cargo engineering, and capability to penetrate physiological barriers. Moreover, attributing to considerable intrinsic immunogenicity, BEVs are able to interact with the host immune system to boost immunotherapy as the novel nanovaccine against a wide range of diseases. Towards these significant directions, in this review, we elucidate the nature of BEVs and their role in activating host immune response for a better understanding of BEV-based nanopharmaceuticals’ development. Additionally, we also systematically summarize recent advances in BEVs for achieving the target delivery of genetic material, therapeutic agents, and functional materials. Furthermore, vaccination strategies using BEVs are carefully covered, illustrating their flexible therapeutic potential in combating bacterial infections, viral infections, and cancer. Finally, the current hurdles and further outlook of these BEV-based nanopharmaceuticals will also be provided.

**Graphical Abstract:**

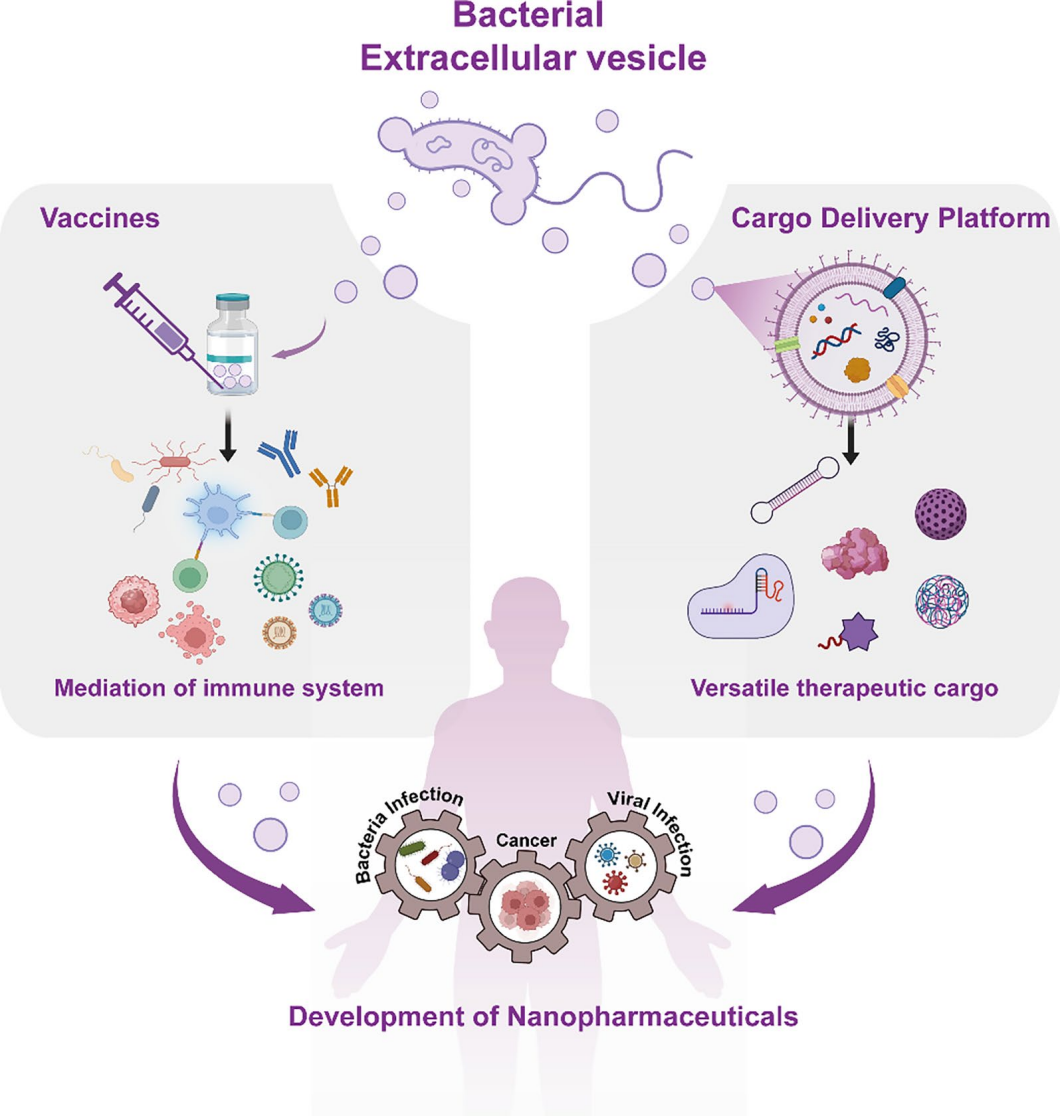

## Introduction

Vesiculation is a crucial and fundamental process across all kinds of species to produce the extracellular vesicles that serve as essential mediators of basic physiological events [1]. These nanovesicles are filled with molecular patterns originated from parent cells, including metabolites, nucleic acids, proteins, and signaling molecules, to maintain cell growth and homeostasis [2, 3]. Biological regulation by extracellular vesicles is widespread across both prokaryotes and eukaryotes [4]. Notably, bacteria, as one of the major inhabitants in the human body, establish intricate relationships with host health and disease, wherein bacterial extracellular vesicles (BEVs) are indispensably involved in these processes [5, 6]. With the increasing and deep understanding of their biological function, BEVs are found to influence various cellular behaviors, including the transport of genetic information, phage infection, mediation of metabolism, as well as interaction between bacteria-bacteria and bacteria-host [7, 8].

Particularly, BEVs are characterized as nanosized nanoparticles, surrounded by lipid-bilayer membranes, ranging from 20 to 400 nm in diameter [7]. In account of the diversity of bacterial types and biogenesis mechanisms, the BEVs could carry versatile cargos inherited from mother cells, such as lipopolysaccharides (LPS), endotoxins, genetic information, cytosolic and membrane proteins [9]. By thanking the unique structure and intrinsic properties of BEVs, these naturally occurring nanovesicles attract the research interest to be developed as novel nanopharmaceuticals, prompting further exploration of their biomedical applications [10, 11]. Generally, BEVs are widely evaluated as biotherapeutics in different forms. Firstly, these nanovesicles with hollow structures could serve as novel drug delivery platforms, facilitating the transport of diverse bioactive molecules and therapeutic cargo to the recipient cells at the lesion site [12]. Thanks to the stability of naturally-occurring membrane structure, the BEVs-based drug delivery platform could carry the therapeutic genetic tools (e.g. siRNA, CRISPR-Cas9, etc.), protecting them from enzymatic degradation or hydrolysis in the complex physiological environment [13, 14]. Besides, benefiting from the ease of modification, BEVs-based drug delivery platforms could efficiently load the small molecular therapeutics [14], or directly produce the synthetic cargo (e.g. antigens, enzymes, therapeutic proteins, etc.) on the BEVs by editing the desired gene in parent bacteria. The BEVs-based drug carriers are also featured in their capability for targeting delivery toward the disease area to enhance drug accumulation and availability [15]. Moreover, the BEVs drug delivery platform can integrate with functional materials, to facilitate combinational therapy (e.g. photodynamic therapy, photothermal therapy, etc.) and maximize the synergistic therapeutic efficiency [16].

On the other hand, due to their similar formulation with parent bacteria membrane, BEVs display abundant pathogen-associated molecular patterns and bacterial membrane antigens, thus endowing unique immunogenicity as the self-adjuvant [17, 18]. Notably, by thanking the immunostimulatory capability of BEVs, these nanovesicles are recognized as powerful and novel components for vaccine development [19, 20]. Typically, the nanosized BEVs can internalize into the immune cells, subsequently inducing a series of therapeutic immune responses. The interaction of BEVs with immune systems can evoke both innate and adaptive immune responses, suggesting the possibility of BEVs to combat infections elicited by bacteria or viruses [21, 22]. Furthermore, BEV-based vaccines have also emerged as attractive platforms for antitumor immunotherapy through the activation of immune cells in tumor regions [21]. Importantly, compared with the traditional vaccination approach, BEVs provide more flexible and universal platforms as the nanovaccine for a broad range of biomedical applications, suggesting their great potential to be developed as a new generation of nanopharmaceuticals [23].

**Fig. 1:**
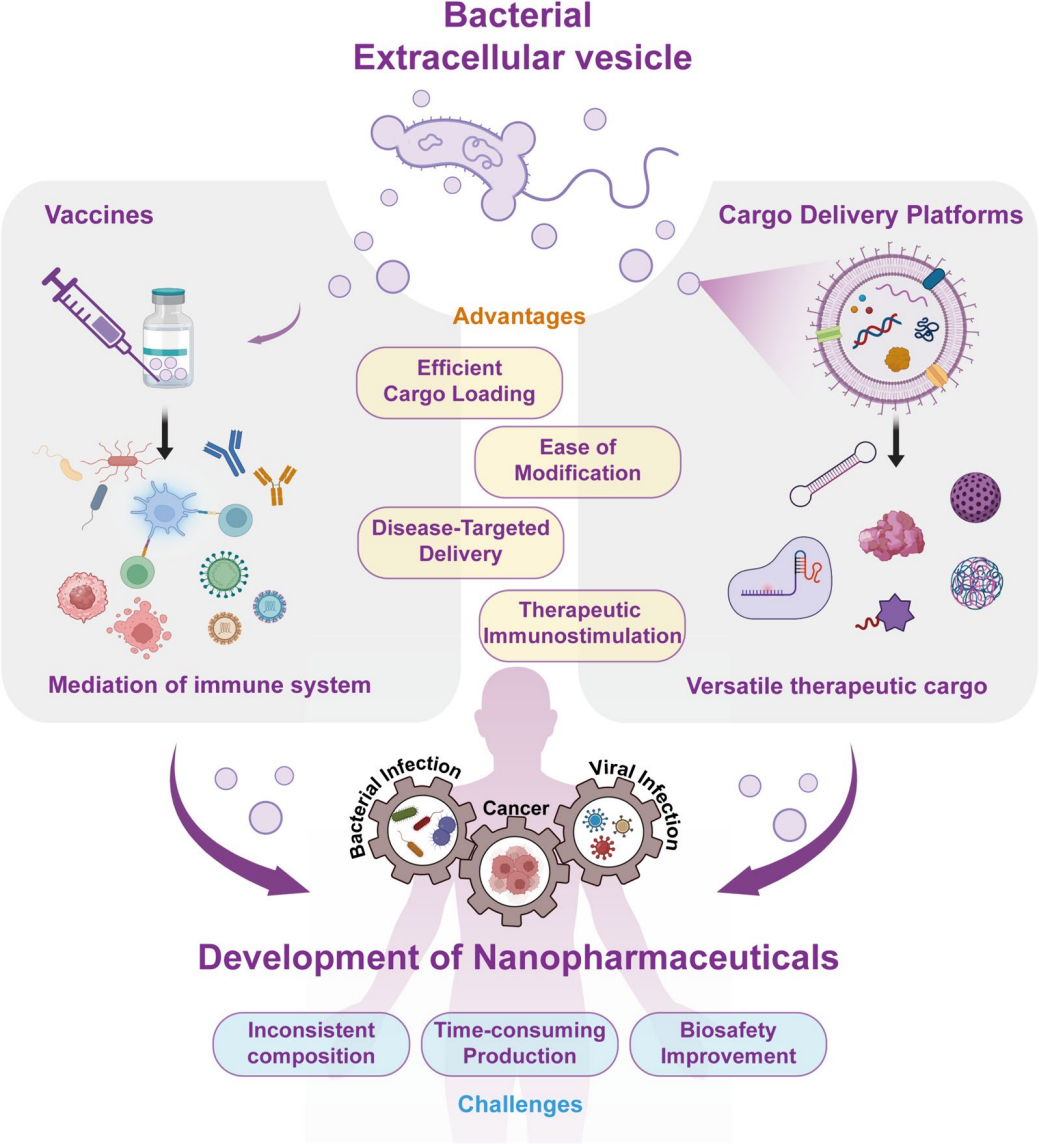
Schematic illustration of developing bacterial extracellular vesicles as new-generation nanopharmaceuticals for biomedical applications, highlighting their unique advantages and addressing the potential challenges in BEV-based nanopharmaceuticals design

In accordance with the promising aspects exhibited by BEVs, this review provides a systematic summary of BEV-based nanopharmaceuticals development for biomedical applications in recent years (Fig. 1). We start with a concise overview of BEVs structure and composition, particularly focusing on elucidating their diverse biogenesis mechanism originating from parent bacteria. Subsequently, we comprehensively demonstrate the interaction of BEVs with host cells to help understand the principles of BEV-based nanopharmaceuticals design. Furthermore, we provide an account of recent advances in BEV-based therapeutics and their biomedical applications, specifically elaborating their utility as drug delivery platforms capable of carrying a range of cargo, such as genetic tools, molecular therapeutics, and functional materials. Meanwhile, we also summarize the noteworthy BEV-based vaccination approaches as powerful platforms to combat various diseases (e.g. bacterial infection, viral infection, and cancer). Finally, we also discuss the current challenge in the development of BEV-based nanopharmaceuticals, aiming to provide meaningful insight for the improvement of novel approaches towards BEVs’ potential clinical practice.

## Biogenesis mechanism of diverse *bacteria*-derived extracellular vesicles

Bacterial extracellular vesicles (BEVs) are crucial biological components mediating the bacteria's cellular events, including nutrient acquisition, genetic information transfer, and mediation of interaction with host cells [7]. All these functions suggest that BEVs exhibit great potential as novel nanopharmaceuticals to combat diverse diseases and regulate healthcare. As a fundamental biological process of living matter, BEVs are produced from parent bacteria by a spontaneous process without additional energy consumption [24]. Thus, a detailed understanding of the structure, composition, biogenesis, and functions of BEVs boosts the development of these naturally-produced membrane entities for biomedical applications, especially as drug delivery platforms and vaccination strategies.

**Fig. 2:**
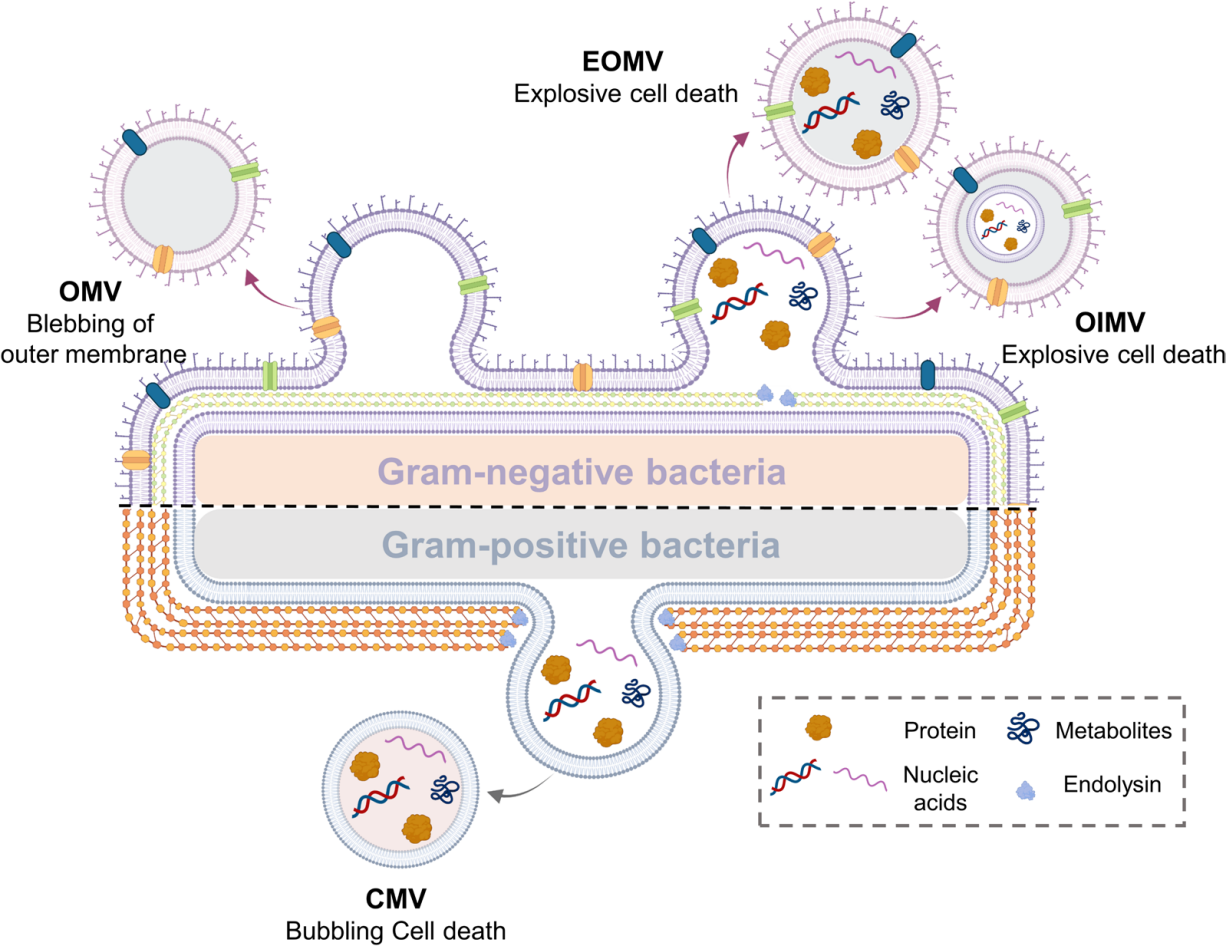
Biogenesis mechanism, Composition, and Classification of Bacterial extracellular vesicles derived from Gram-positive or Gram-negative Bacteria

Due to the different types of parent bacteria, including Gram-negative and Gram-positive strains, the diverse biogenesis mechanisms lead to the unique membrane structure and loaded contents of distinct BEV types. Typically, Gram-negative bacteria have a double-layered membrane structure, comprised of the outer membrane, periplasmic space, and cytoplasmic membrane, while Gram-positive bacteria have only one cytoplasmic membrane covered by a thick cell wall of peptidoglycan [25]. The various formation mechanisms and characteristics of BEVs are attributed to their original bacteria as shown in Fig. 2.

The extracellular vesicles derived from Gram-negative bacteria have two main models of their biogenesis mechanism, including blebbing of the outer membrane and explosive cell lysis. When the cell envelopes suffer from abnormal disturbances (e.g. hydrophobic compound interaction, instability of peptidoglycan biosynthesis, denaturation of membrane protein, etc.), the outer membrane will undergo the blebbing to produce the outer membrane vesicles (OMVs) [7, 9]. During this progress, the inner membrane remains intact to prevent loading the cytoplasmic cargo into the OMVs. The size of OMVs is around 20–250 nm with spherical morphology, containing abundant lipopolysaccharides (LPS), lipids, and membrane proteins [24, 26]. On the other hand, when the peptidoglycan layer of Gram-negative bacteria is weakened by autolysin, the inner membrane will subsequently protrude into the periplasm to produce the outer-inner membrane vesicles (OIMVs). Similarly, the explosive outer membrane vesicles (EOMVs) were produced by the model of explosive cell death model [7, 27, 28]. The phage-derived endolysin destroys the peptidoglycan layer around the bacteria, inducing the cell explosion and the shattered membrane fragments fuse to generate the EOMV. OIMVs are comprised of two lipid bilayers, the outer membrane and inner membrane from the parent bacteria as well as the membrane proteins, while the EOMV only has one lipid bilayer from the original outer membrane. Both EOMV and OIMV contain cytosolic cargo (e.g. small-molecule metabolites, genomic DNA, RNA, endolysin, virulence component, etc.) that differ from the OMVs [29].

Besides, in the specific Gram-positive bacteria, endolysin will initiate the bubbling cell death by hydrolysis of the thick peptidoglycan cell wall, potentiating the formation of cytoplasmic membrane vesicles (CMVs) [7]. CMVs formation is attributed to the stress-mediated bacteria lysis, peptidoglycan degradation by exogenous endolysins, and drug-induced suppression of cell wall biosynthesis. CMVs also contain the cytosolic cargo from Gram-positive bacteria, similar to the EOMVs and OIMVs [30]. Notably, the different composition and structure of BEVs resulted in distinct biological functions in the physiological environment, especially for their interaction with the host immune system [31]. Hence, the BEVs attracted great interest from academia and industries to be utilized as nanopharmaceuticals for biomedical applications, especially for drug delivery and vaccination to combat various diseases.

## Internalisation of BEVs into host cells

**Fig. 3:**
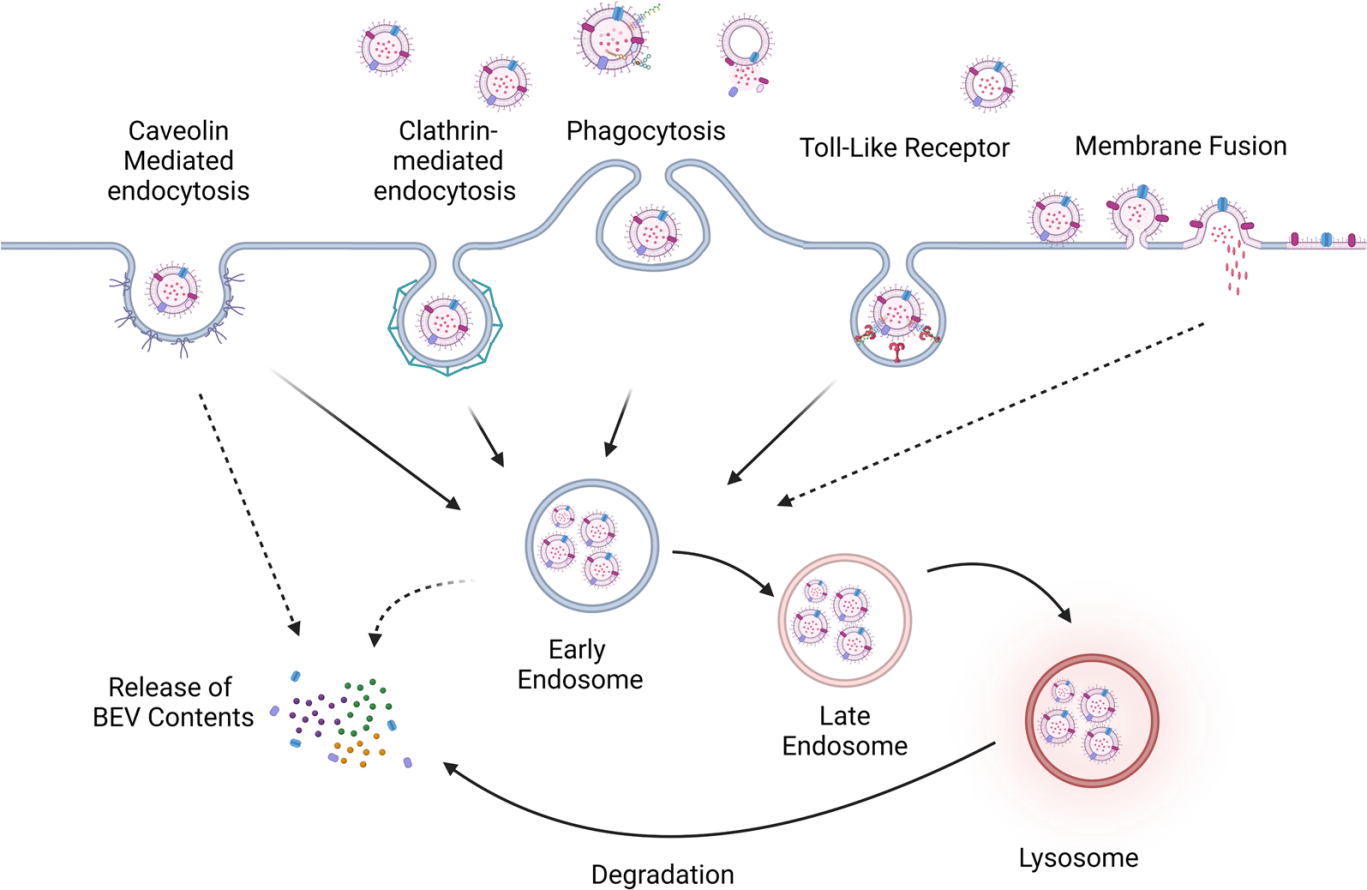
Major internalization pathways taken by BEVs into host cells. Depending on their nano size, the type of recipient host cell, and the ligand-receptor interactions triggered, BEVs are internalized via caveolin and clathrin-mediated endocytosis, phagocytosis, toll-like receptors, and membrane fusion

As key messengers for microbiota-host communications, BEVs carry a wide range of cargoes such as proteins, DNA, and RNA. The majority of these “messages” are compartmentalized inside BEVs and are required to be released into the host cells to facilitate cellular events [32, 33]. For this to happen, BEVs need to enter host cells and ultimately release these cargoes untainted for them to fulfill their biological roles. Depending on their origination, the exterior of BEVs is decorated with ligands such as LPS, lipoproteins, and other virulent factors [34]. These ligands play an important role in the internalization of BEVs, as their interaction with different receptors on the host cell triggers different internalization pathways [35]. The internalization pathway taken by BEVs is also influenced by the size of BEVs [36], the type of host cells along with many other unknown factors [37]. The internalization processes of BEVs by host cells remain underexplored, but a few major pathways have been proposed and studied (Fig. 3).

The most common internalization pathway utilized by extracellular entities such as BEVs is phagocytosis, particularly for entry into phagocytotic immune cells such as neutrophils, dendritic cells, and macrophages [38]. Notable examples of BEVs internalized via phagocytosis are *Streptococcus pneumoniae* [38] and *Mycobacterium tuberculosis* [39, 40]. Phagocytosis is initiated by phagocytic receptors, which trigger a signaling cascade leading to the rearrangement of lipid membranes and the actin cytoskeleton [40]. Phagocytosis is initiated by phagocytic receptors, which triggers a signaling cascade leading to the rearrangement of lipid membranes and the actin cytoskeleton, ultimately resulting in the surrounding of the BEVs [41], eventually forming phagolysosomes in which the BEVs may be degraded to release their internal cargo [42].

BEVs are also found to enter non-phagocytotic cells such as epithelial cells, suggesting that many other non-phagocytotic pathways are possible [35]. An example of such pathways is Clathrin-mediated endocytosis (CME) [43, 44]. The initiating ligands and relevant receptors are poorly understood, but the formation of clathrin-coated pits has been well studied. The first proteins that assemble at the site of BEV docking are clathrin, which is followed by an assortment of structural proteins to form a clathrin-coated pit. Dynamin2 is further recruited at the neck of the developing invagination, which undergoes GTP hydrolysis-dependent conformational changes to cut off the nascent intracellular vesicle [45]. The BEV-carrying intracellular vesicles fuse with endosomes and eventually release their cargoes upon disintegration of the BEV lipid bilayer. This pathway is exclusively taken by BEVs of *Lactiplantibacillus plantarum* [46], where the uptake of the BEV was blocked upon treatment with CME inhibitor chlorpromazine. However, the internalization of BEVs may not rely solely on one pathway; instead, multiple pathways can be simultaneously utilized. As in the case of *Borderella bronchiseptica* [43], their internalization into AW264.7 cells was decreased upon the inhibition of either micropinocytosis with cytochalasin D and CME, suggesting that the BEVs rely on both micropinocytosis and CME for internalization.

BEVs along with pathogens such as viruses and bacteria, can invade host cells through receptor triggered-internalization pathways such as Caveolin-mediated endocytosis [47]. This is a preferred pathway as the resulting intracellular caveolae are believed to not fuse with lysosomes [48, 49] hence ensuring the survival of the invading pathogens. Caveolin-mediated endocytosis is initiated with the binding of ligands and virulent factors like folic acids [50], alkaline phosphatase [51], cholera toxin [51], and viruses like HIV1 [52]. This is followed by oligomerization of caveolin proteins on lipid raft domains to form the flask-shaped invaginated caveolae [53]. The caveolae pinch in a GTP-dependent manner similar to Clathrin-coated pits to form caveosomes, whose intracellular fate depends on their content [49]. Caveosomes containing the SV40 virus were found to have neutral pH and do not fuse with lysosomes [54], while albumin-rich caveosomes were trafficked along the endosomal degradation pathway [55]. It can be inferred that the interactions between cargo and caveolar components play a role in the destination of caveosomes [49]. In the works of Franz G. Zingl, outer membrane protein (Omp) OmpU and OmpT were found to be essential for the predominant caveola-mediated internalization of *V. cholerae* BEVs [56]. These BEVs protected the virulent factor cholera toxin (CT) from extracellular trypsin and successfully releasing them in HT29 cells. This reiterates the importance of surface ligands in the initiation of Caveolin-mediated endocytosis which allows successful delivery of cargoes into host cells.

The endocytosis pathways mentioned earlier involve the coating of the entire BEV including its lipid bilayer, with transmembrane proteins like Caveolin to form intracellular vesicles. However, there are also pathways taken by BEVs in which only the internal cargo is internalized without the lipid bilayer of the BEV. One such pathway is Membrane fusion, which is a process triggered by the binding N-ethylmaleimide-sensitive factor (NSF)-attachment protein receptors (SNAREs) [57]. This pathway requires the presence of Ca^2+^ ions and it is observed that during fusion, the phospholipid bilayers of BEVs adhere and assimilate with host cell [57]. This is well demonstrated by Bomberger et al., where the fluorescence of Rhodamine‐R18 membrane-labelled *P. aeruginosa* BEVs were increased when treated to mammalian epithelial cells, showing a mixing of lipids between the two bilayers and eventual dilution of the previously quenched dye [58].

Another internalization pathway taken by BEVs is through Toll-like receptors (TLR), a family of cellular receptors that recognizes microbial molecules [59]. TLRs constitute the primary strategy for the detection of xenogeneic substances, such as the detection of LPS of Gram-negative bacteria by TLR4 or Lipopeptides of Gram-positive bacterial cells via TLR2[60]. These TLR-detectable ligands are an integral part of BEVs. Upon binding of BEVs to TLRs, a cascade of signal events occurs, leading to the internalization of the BEV-TLR complex into the cell as endosomes [61], which may further develop into autophagosomes for BEVs disintegration and release of cargoes. For example, BEVs secreted by *Staphylococcus Aureus* containing immunostimulatory cargoes are internalized by lung epithelial A549 cells via TLR2 and induced autophagy [62], while BEVs of Gram-negative bacteria *Bacteroides thetaiotaomicron* were predicted *in sillico* and experimentally confirmed to be internalized via the TLR4 pathway [63].

Based on their origination and surface ligands, BEVs can interact with different receptors on the membrane of host cells and trigger a variety of internalization pathways for their entry. The internalized BEVs’ phospholipid bilayers can be disintegrated in late endosomes or lysosomes to release their internal cargo for pathogenic or therapeutic effects. In some cases. The mere interaction of BEVs with host cell surface receptors is sufficient to trigger other cellular events such as immune responses without having to enter the host cell [64].

## Interaction of BEVs with the host immune system

**Fig. 4:**
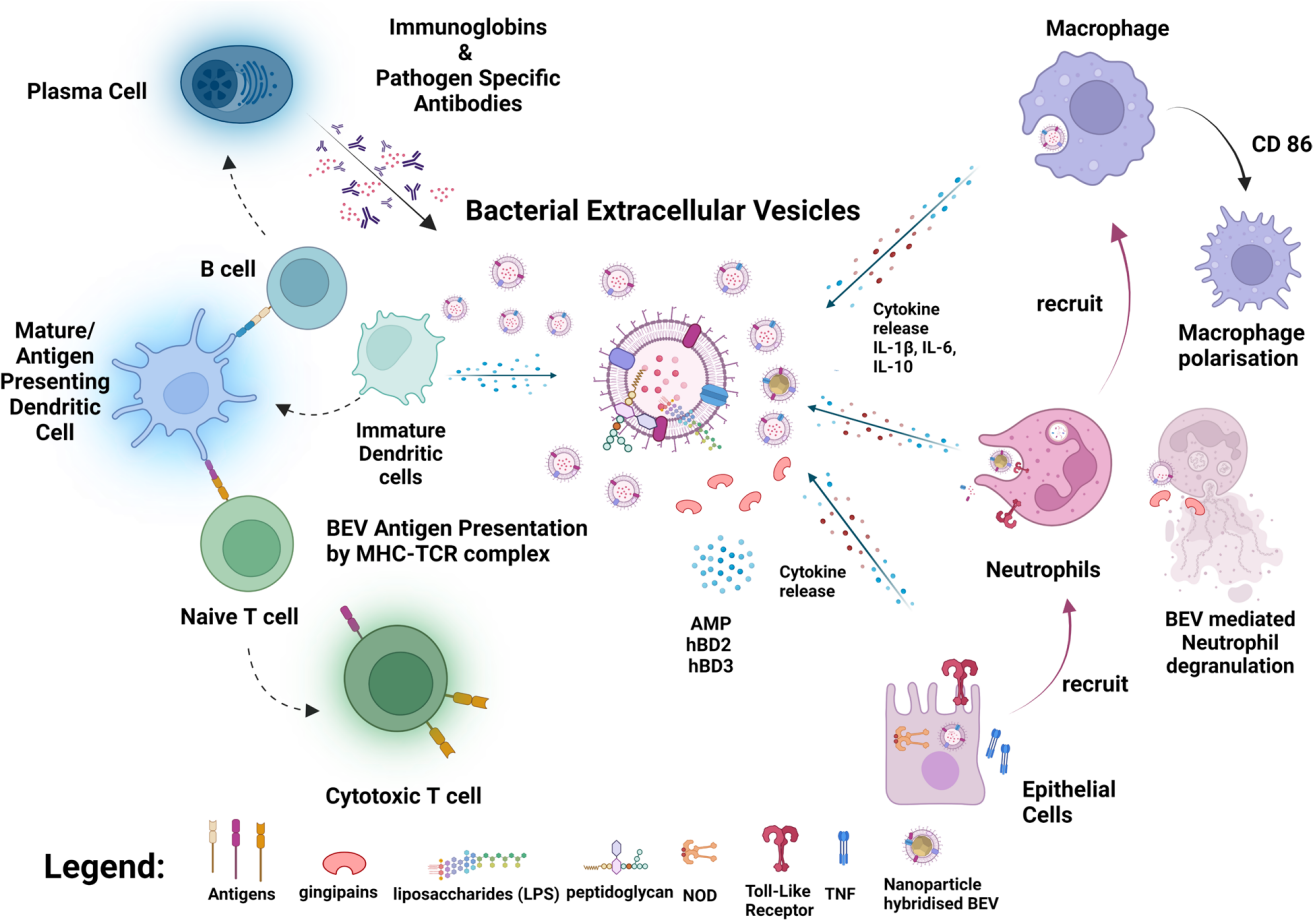
The interactions of BEVs with various cellular components from the adaptive and innate immune response

As BEVs are isolated from bacteria that maybe originally pathogenic or probiotic, they retain the pathogen-associated molecular patterns (PAMPs) of their parent bacteria factors such as liposaccharides (LPS), peptidoglycan, or DNA [65]. These immunostimulatory biomolecules are recognized by pathogen recognition receptors (PRRs), which can be found on epithelial [66] and immune cells [67], triggering the innate and adaptive immune response. The interactions between BEVs and different components of the immune system will be discussed in this section (Fig. 4).

Upon an invasion of bacteria and their BEVs, epithelial cells serve as the frontline defenders as they are usually the first obstacles met by invading pathogens [68]. Although epithelial cells are not strictly immune cells, they are armed with PPRs such as TLRs [69], nucleotide-binding oligomerization domain–like receptor (NOD) [70]. Meanwhile, these cells are capable to release cytokines to stimulate innate and adaptive immune responses upon detection of BEVs. Indeed, the BEVs of *Porphyromonas gingivalis* [71] inherited several virulent factors such as LPS and gingipains (cysteine proteinases), inducing the production of pro-inflammatory cytokines, interleukin (IL)-6, and IL-8 in human gingival epithelial cells. Furthermore, these BEVs can travel in the bloodstream from their site of infection in the mouth to distal organs such as the lungs, showing that BEVs play a role in systemic *Porphyromonas gingivalis* infection. Similarly, the BEVs of *Fusobacterium nucleatum* stimulated the secretion of interleukin-8 (IL-8) and tumor necrosis factor (TNF) in colonic epithelial cells [72]. These secretions were perturbated by the treatment of TLR4 inhibitors, suggesting that transmembrane TLR4 activation was required in this proinflammatory signaling. As most BEVs are internalized into host cells as mentioned earlier, they can also activate intracellular receptors in epithelial cells, such as NOD1. Gram-negative mucosal bacteria *Helicobacter pylori, Pseudomonas aeruginosa, and Neisseria gonorrhea* secreted BEVs containing peptidoglycans that can trigger intracellular receptor NOD1 cell signaling which subsequently release IL-8 and antimicrobial peptides (AMP) human-β-defensin 2 (hBD2) and hBD3 [73]. This study shows that BEVs can be detected by intracellular receptors and epithelial cells can participate directly in immune response against BEVs other than mere recruitment of immune cells via release of cytokines.

The first true members of the immune system to respond to bacterial infections and the release of BEVs are the neutrophils. They can be recruited to infection sites by cytokines such as CXCL1/IL-8, released by endothelial cells upon TLR4 interaction with *E. Coli* BEVs [74]. In another instance, BEVs of *Haemophilus influenzae* induced airway epithelial cell secretion of IL-1β, which further induced Th17 cells to release IL-17 to recruit neutrophils [75]. As part of the innate immune system, neutrophils are phagocytotic cells that internalize invading pathogens like bacteria [76]. Although is it unclear whether neutrophils can internalize pure BEVs, current studies are showing *E. Coli* BEVs hybridized with nanoparticles can be internalized and hitchhike on neutrophils as a chemotaxi [77, 78]. Neutrophils via a whole arsenal of antimicrobial agents in cytosolic granules [79], eradicate invading pathogens effectively, but this pathway can be countered by BEVs of *Porphyromonas gingivalis* [80], which can degranulate neutrophils and secrete gingipains, which are proteases that will cleave antimicrobials agents such as myeloperoxidase (MPO) and LL-37, sustaining immuno-evasion. Other than phagocytosis of pathogens, neutrophils can also secrete cytokines and chemokines for further reinforcement of other immune cells to fight against infections [81]. Upon triggering by the BEVs of N. meningitidis, neutrophils released tumor necrosis factor (TNF)-α, IL-1β, facilitating inflammation [82]. Furthermore, macrophage inflammatory protein-α and macrophage inflammatory protein-β are also secreted, which recruits macrophages for immune reinforcement [83].

Macrophages are integral to the innate immune response against pathogens, actively phagocytosing them and simultaneously secrete cytokines and antimicrobial agents, for the enhancement of antimicrobial effects [84]. The BEVs of *E. Coli* and *S. Aureus* were quickly taken up by macrophage cells in vitro [85, 86]. This increased the expression of cytokines (such as IL-1β, IL-6, IL-10) and costimulatory molecules (CD 86) in macrophages, effectively turning polarising them from M0 to the pro-inflammatory M1 phenotype.

Not only are BEVs responded with a short-term immune response, but studies have also shown that BEVs are also capable of stimulating the adaptive immune response, leading to long-lasting resistance against such BEVs (Fig. 4). This is achieved via the maturation of dendritic cells, which present pathogenic antigens to T and B cells for their activation [87, 88]. The BEVs of *Salmonella typhimurium* stimulated the maturation of DCs with increased expression of MHC-II and CD-86 which subsequently led to the activation of T and B cells for immunity against live bacteria challenges [89]. When bone marrow-derived dendritic cells (BMDCs) are treated with the BEVs of periodontal pathogens *Porphyromonas gingivalis* and *Tannerella forsythia,* the expression of cytokines IL-1β, IL-6, IL-23, and IL-12p70 were triggered. This was not observed for *Treponema denticola* [90]. It was elucidated to be due to the proteolytic capabilities of the BEVs, which degraded the secreted cytokines. By coculturing Naïve CD4 + T cells with such BEV-primed BMDCs, the T cells were differentiated differently, where *P. gingivalis* and *T. denticola* led to the induction of IL-17A^+^ T cells whereas *T. forsythia* largely induced interferon (IFN)-γ^+^ T cells with IL-17A^+^ T cells as the minority. Treatment of BEVs of *E coli* in MC38-OVA and B16F10-OVA tumor mice models also recruited cancer antigen-specific CD8 + T and increased their expression of cytotoxic molecules such as granzyme B, TNFα, perforin, IFN-γ [91].

B cells are also key role players in developing adaptive immunity against pathogens, producing bactericidal antibodies, and forming memory cells for prolonged and acute responses against future infections [92]. BEVs of *Neisseria meningitidis* were reported to stimulate B cells to produce cross-reactive antibodies that were bactericidal towards both *Neisseria meningitidis* and *Neisseria gonorrhoeae* [93]*.* This was possible as the 2 pathogens display similar antigens (PorA/B and lipooligosaccharide) on the whole cell and their BEVs. Meanwhile, the BEVs of *Neisseria lactamica* OMVs could induce tonsillar B cells to produce polyclonal IgM and increase the proliferation of a subset of B cells, through a mechanism that is possible via a mitogenic ligand and B cell receptor interaction [94]. Mice injected with BEVs of *Salmonella typhimurium* displayed high amounts of antigen-specific IgG produced by B cells and were protected against live *Salmonella typhimurium* challenge 14 days after injection, suggesting an established adaptive immunity [89]. Studies on *Neisseria meningitidis* BEV-based vaccines show that memory B cells were activated and isolated upon vaccination [95, 96].

The interactions between BEVs and various components of the immunity systems to elicit innate and adaptive responses entail the potential of using BEVs as vaccines to induce immunity against future infections. It is potentially safer to utilize BEVs as vaccines as compared to attenuated cells or whole cells as BEVs themselves are not propagative and thus will not lead to serious infections/adverse reactions, as opposed to when using live bacteria [97].

## Recent advances in BEVs-based nanopharmaceuticals

### BEVs as drug delivery platform

#### BEV delivery of gene therapy

In recent years, a new form of pharmacological approach to treating disease is Gene therapy. Gene therapy involves the introduction of genetic materials such as DNA or RNAs and enzymes like nucleases or genome editing enzymes [98]. These materials are introduced into host cells often for gene silencing (using miRNA, siRNA, and shRNA), gene introduction via plasmids, naked genetic materials, and gene editing using nucleases or clustered regulatory interspaced short tandem repeats (CRISPR)/CRISPR-associated protein (Cas)-associated nucleases [99]. Gene therapy has emerged as a potent treatment modality, with several genetic-based treatments demonstrating clinical success [100]. Unfortunately, genetic materials and their associated enzymes are highly susceptible to degradation both ex vivo and in vivo, rendering them suitable for direct introduction into hosts [101]. Furthermore, these materials lack targeting capabilities and can be internalized by non-target cells leading to adverse physiological effects [102]. Thus, gene therapy is often actualized by the loading of these genetic materials into nanocarriers such as viral capsules, liposomes [13], exosomes [103], and synthetic nanoparticles [104, 105]. These nanocarriers provide protection of their genetic cargo from the physiological conditions and enable directed delivery to target sites [106, 107]. BEVs can also be potential carriers for gene therapy, as BEVs from *E coli* and *H. pylori* respectively have been found to contain genetic materials for the development of antibiotic resistance [108] and regulation of host immune responses [109]. In comparison to the other nanocarriers, BEVs boast greater penetrative capabilities, ease of modification, and most importantly, the potential to be able to be industrially mass-produced [16]. Indeed, several advances utilizing BEVs as a delivery platform for the administration of gene therapy have shown preliminary success.

**Fig. 5:**
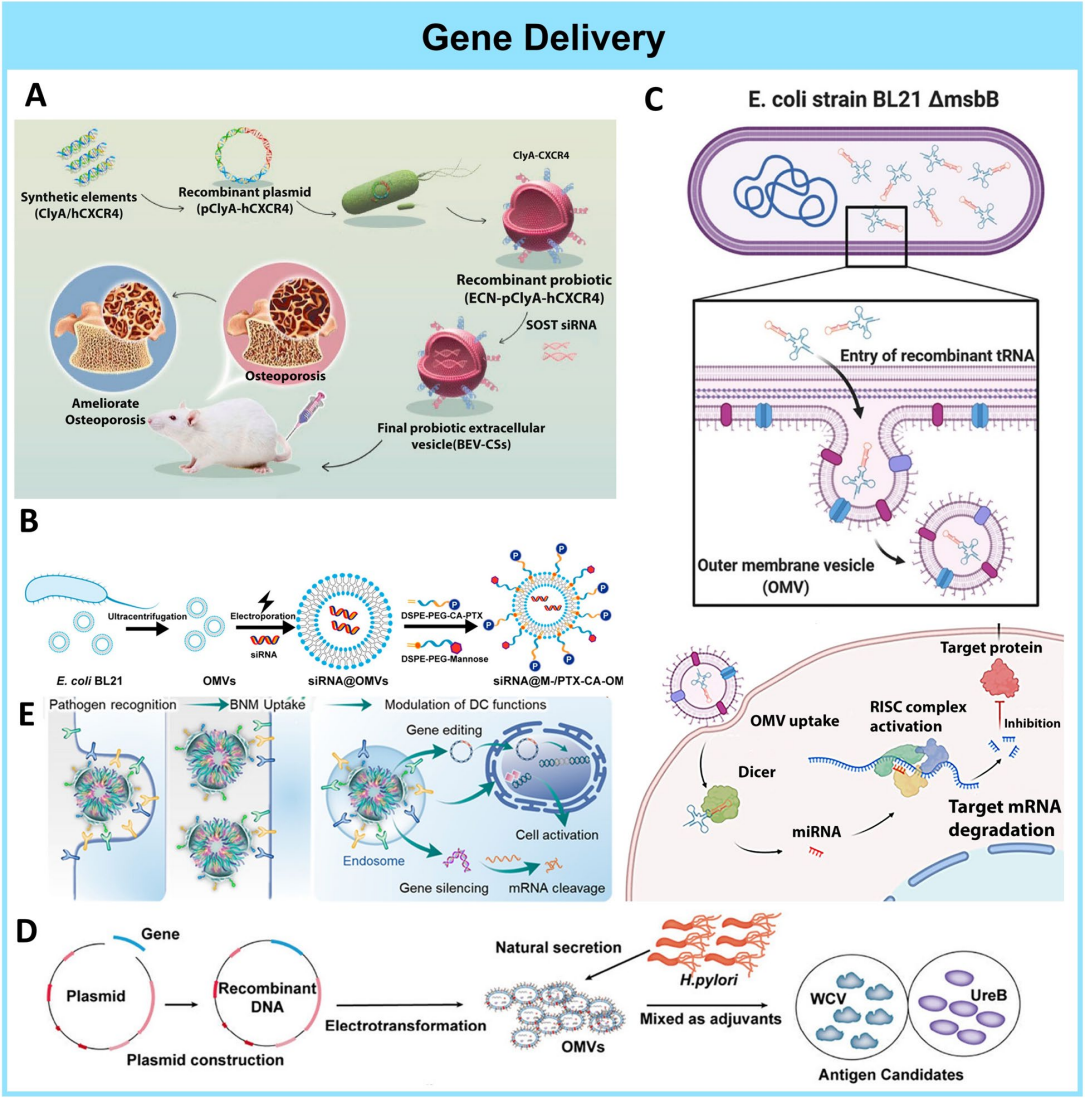
BEV as gene delivery platforms. **A.** Schematic of Bone targeting BEVs-hCXCR4-SOST iRNA (BEVs-CSs) for osteoporosis treatment. Reproduced with permission. [110] Copyright 2019, American Chemical Society **B.** Schematic of siRNA@M-/PTX-CA-OMVs for modulation of macrophage metabolism and tumor metastasis suppression. Reproduced with permission. [113] Copyright 2021, American Chemical Society **C.** Schematic of OMV^tRNA−pre−miR−126^ against breast cancer. Reproduced with permission. [117] Copyright 2022, Elsevier. **D.** Schematic of BEV-delivered DNA plasmids as vaccines. Reproduced with permission. [119] Copyright 2023, American Society for Microbiology. **E.** BEV delivered CRISPR-Cas 9 for dendritic cell-targeted gene editing. Reproduced with permission. [120] Copyright 2023, American Chemical Society

The exemplary targeting capabilities of BEVs are particularly highlighted in the study conducted by Han Liu et al., BEVs derived from probiotic *E. Coli* Nissle 1917 loaded with siRNA, were applied in the amelioration of osteoporosis (Fig. 5A) [110]. The treatment of osteoporosis is traditionally achieved by hormonal drugs which are often plagued with side effects [111]. Genetic therapy using siRNA to silence the SOST subsequent inhibition of the WNT pathway has previously been demonstrated to promote bone formation and ameliorate osteoporosis. But the short half-life and poor penetrations of these siRNAs have limited their success [111]. To overcome these limitations, engineered *E. Coli Nissle 1917* displaying fused C-X-C motif chemokine receptor 4 (CXCR4) on the outer surface and internally loaded with SOST siRNA were developed. The fusion of hCXR4 with ClyA membrane protein facilitated the surface expression of hCXR4, endowing the BEVs with bone-targeting capabilities. This modification successfully escorted the SOST siRNA to the femur bones of mice. Mice treated with these BEVs exhibited higher bone mass and improved microarchitecture. This study on bioengineered-BEV-based genetic therapy demonstrated that BEVs can be readily modified to incorporate non-native targeting capabilities, thereby serving as an effective delivery platform for siRNA therapies.

As previously mentioned, BEVs possess immunogenic properties due to the Pathogen-Associated Molecular Patterns (PAMPs) expressed on their surfaces. This characteristic enables them to be readily recognized and taken up by macrophages compared to other exosomes [112]. This property is exploited in the works of Qin Guo et al., for the targeting of Tumour Associated Macrophages (TAM) and tumor metastasis suppression [113]. In metastatic tumors, upregulation of Redd1 inhibits macrophage glycolysis, which is initiated by tumor cell signaling. Combining tumor cell killing with the Redd1 shutdown could effectively inhibit solid tumor metastasis. Therefore, a nanocarrier system utilizing *E. Coli* BL21, capable of pH-responsive release of Paclitaxel and delivery of Redd1 siRNA, was developed (Fig. 5B**)**. Upon treatment of the nanocarrier system to tumors, surface-anchored Paclitaxel would be released initially upon reaching the tumor site, exerting cytotoxic effects on tumor cells, and stopping tumor cell-initiated Redd1 upregulation. The Redd1 siRNA-carrying nanocarrier would be sequentially uptake by TAMs to restore their glycolysis levels and polarise them into a tumor progression-inhibiting phenotype. This study demonstrates how the affinity of BEVs to immune cells was utilized for combinational gene therapy with small organic molecules and inspires future work of using BEVs for immune cell targeting.

MicroRNA (miRNA)-mediated gene therapy has emerged as a promising tool to combat cancers through the regulation of target genes in tumor cells [114]. Recent development have utilized pre-miRNA instead, which are shorter precursors of miRNA that can be processed into mature miRNA for RNA interferences [115]. However, these approaches are still plagued by synthetic difficulties, degradation by intracellular nucleases, and poor loading into nanocarriers which are often toxic [116]. Inspired by these challenges, Cui et al. developed a msbB mutated *E. Coli* BL21 derived BEVs carrying tRNA^Lys^-pre-miR-126 (Fig. 5C) [117]. The unique cargo is a pre-miRNA that is disguised with a “tRNA scaffold”, which is more stable and can be amplified into mature miRNAs in host cells [118]. The highly biocompatible BEVs targeted tumor cells in mice specifically with the AS1411 aptamer on their surfaces and lowered the expression of CXCR4, effectively lowering tumor proliferation. This study shows that BEVs can be bioengineered to be superior and safe nanocarriers that can accommodate non-conventional genetic cargoes.

In addition to delivering various forms of RNAs for gene therapy, BEVs can also serve as carriers for DNA, acting as adjuvants for vaccines. In the works of Qiong Liu et al., DNA was delivered in the form of eukaryotic expression plasmid coding for cytokines IL17A or INF-γ in *H. pylori* BEVs (Fig. 5D) [119]. The recombinant BEVs were used in conjunction with UreB and whole inactivated cells as the vaccine antigen. Mice injected with the BEV adjuvants had higher levels of anti-*H. pylori* IgG antibodies and a more lasting expression of IL-17A and IFN-γ than control groups. When challenged with *H. pylori* infection, mice immunized with the recombinant BEVs adjuvants showed lower *H. pylori* colonization than wild-type *H. pylori* and Chlorea Toxin as adjuvants. Overall, the recombinant BEVs were able to induce stronger humoral, and mucosal immune responses and protected hosts from *H. pylori* infections. This study serves as an example demonstrating that BEVs can effectively deliver DNA cargo, acting as adjuvants for vaccine development.

In addition to gene silencing or gene introduction, gene editing through Clustered Regularly Interspaced Short Palindromic Repeats (CRISPR)-Cas9 has proven to be a powerful method for permanently knocking out genes [121]. However, guide RNAs and CRISPR-Cas 9 enzymes used in CRISPR-Cas-9 gene therapy are susceptible to degradation by nucleases and their poor penetration and selectivity into target cells have limited their application as a viable treatment modality [122, 123]. To fully draw upon the prowess of CRISPR-Cas 9 gene editing, Min Li et al. developed a novel bacterial nanomedicine (BNM) based on polymer-lipid hybrid nanoparticles and BEVs from attenuated *Salmonella* to deliver CRISPR-Cas 9 to Dendritic Cells [120] (Fig. 5E). The BEV-derived LPS conferred Dendritic Cell (DC) targeting capabilities to the Biomimetic Nanoparticles (BNMs) through TLR4-PAMPs interactions. BNMs with higher BEV to NP ratios exhibited increased uptake by DCs, consequently leading to elevated expression of costimulatory molecules CD80, CD86, and CD40 in these DCs. The CRISPR-Cas9 system delivered by these BNMs successfully knocked out the YTHDF1 gene in DCs, leading to CD8 + T cell-mediated tumor inhibition in MC38 tumor-bearing mouse models. The targeted delivery of sensitive RNAs and CRISPR-Cas9 specifically to dendritic cells (DCs) for DC activation and tumor inhibition underscores the significant potential of BEVs as a versatile delivery platform for effective gene therapies.

#### Therapeutic molecular cargo

The use of small molecules and biologics for the treatment of diseases has always been the mainstream modality since the dawn of modern medicine [124]. Despite their extensive use as therapeutics against diseases, they also suffer limitations as well, such as non-selectivity leading to adverse side effects and susceptibility to metabolism in vivo [125, 126]. As mediators between bacterial interspecies communication or bacteria–host interkingdom interaction, BEVs have intrinsic targeting capabilities that can bring chemical/biological messengers to the intended destination [127]. These properties can be hijacked by loading small molecules or biological drugs into specially engineered BEVs, in which they can be delivered specifically to the site of infection/disease. This results in the accumulation of therapeutic molecules at the target site, enhancing the therapeutic effect and reducing adverse effects due to off-target interactions [124]. Moreover, the loading of drugs into BEVs protects them from metabolism or degradation during their circulation to the target site. Consequently, this enhances the effective drug concentration at the target site and mitigates toxicity issues associated with drug metabolism [128]. Thus, there have been many advances utilizing BEV-based drug delivery platforms to overcome the limitations of small molecule and biological drugs [124, 129].

**Fig. 6:**
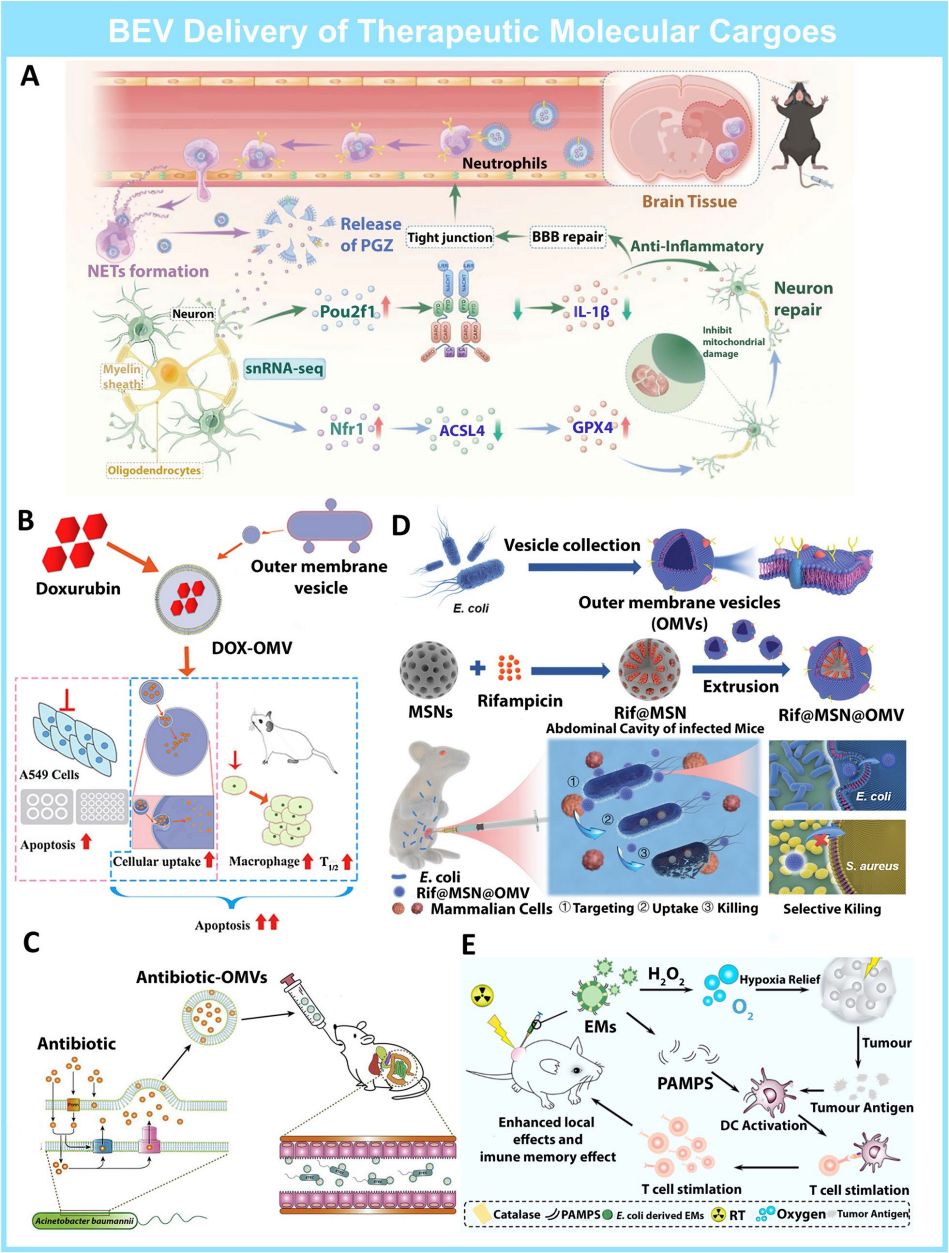
BEVs used as delivery platforms for therapeutic molecular cargo **A.** Schematic of OMV@PGZ Hitchhiking neutrophils for Enhanced Ischemic Stroke Therapy. Reproduced with permission [134] Copyright 2023, John Wiley and Sons **B.** Schematic of BEV delivery of Doxorubicin for chemo-immunotherapy. Reproduced with permission [140] Copyright 2020, Elsevier Inc. **C**. Schematic of Levofloxacin loaded BEVs against antibiotic-resistant bacteria. Reproduced with permission [143] Copyright 2020, Elsevier Inc. **D**. Mesoporous silica BEV hybrid nanosystem delivery of rifampicin to overcome antibiotic resistance. Reproduced with permission [146]. Copyright 2021, Wiley–VCH **E**. Schematic of BEV delivered Catalase relieves tumor hypoxia and enhances radiotherapy. Reprinted with permission [148]. Copyright 2021, American Chemical Society

The small size and interactive nature of host cells [130] of BEVs enable them to cross multiple membranes and physical barriers in the human body. An example of a membrane barrier is the blood–brain barrier (BBB), which divides the brain from the peripheral circulation, maintaining homeostasis and the brain microenvironment [131]. Its highly lipophilic nature prevents 98% of drugs from reaching the brain and exerting their intended therapeutic effects [132]. Neutrophils were reported to be able to pass through the BBB and their expression of TLRs facilitates the interaction and uptake of BEVs [133]. Based on these desirable properties of neutrophils, Pan et al. loaded pioglitazone *E. Coli* BEVs with (OMV@PGZ), which would hitchhike onto neutrophils to cross the blood–brain barrier to enhance Ischemic Stroke Therapy (Fig. 6A) [134]. Neutrophils can engulf OMV@PGZ within 90 min, effectively cross the BBB, and release the intracellular OMV@PGZ after arriving at the ischemic area, where excess reactive oxygen species (ROS) induces the disintegration of neutrophils to form neutrophil extracellular traps (NETs). The release of PGZ in ischemic sites activates peroxisome proliferator-activated receptor (PPARγ), which ultimately exerts neuroprotective effect in transient middle cerebral artery occlusion (tMCAO) mice models. In this study, the unique immunogenicity between BEVs and neutrophils was taken advantage of to deliver small molecular drugs across the BBB.

BEVs have unique targeting abilities that can be utilized to load and deliver therapeutic drugs to disease sites. Chemotherapy drugs such as Doxorubicin are used as frontline drugs in the treatment of various cancers [135]. However, the non-selective nature of Doxorubicin (DOX) has resulted in severe adverse effects such as myelosuppression and immunosuppression, greatly reducing their application in the clinical setting [136, 137]. Previous studies have attempted to address selectivity issues by loading DOX into liposomes. However, these liposomal themselves are plagued with toxicity issues as well [138]. BEVs are superior alternatives to liposomes as they are highly biocompatible, and their outstanding immunogenicity may induce a local immune response which may augment the anti-tumor effects of DOX [139]. This is exemplified in the works of Kuerban et al., who loaded DOX into attenuated *Klebsiella pneumonia* BEVs (DOX-OMV) as a treatment for non-small-cell lung cancer (NSCLC) (Fig. 6B) [140]. Compared to free DOX, DOX-OMVs displayed superior pharmacokinetic properties, higher uptake, and a more potent cytotoxic effect in A549 cells. Interestingly, the empty BEV carrier exhibited a more potent antitumor effect than free DOX and DOX-liposomes, which was suggested to be contributed by the BEV-induced accumulation of macrophages at the tumor site. In another example, Zhuang et al. constructed the *E. Coli*-derived OMVs by encapsulating the inhibitors (UNC2025) of myeloid-epithelial-reproductive tyrosine kinase (MerTK) to block the efferocytosis of apoptotic tumor cells [141]. Subsequently, the released tumor-associated antigens will be covalently linked to the OMVs and delivered to the lymph nodes, evoking the maturation of dendritic cells and boosting the immunotherapy combating the xenografted, metastatic as well as recurrent tumor models of mice. This study demonstrates that other than merely serving as a drug delivery platform, BEVs can also serve as an immune-response stimulant for synergistic anticancer therapy.

With their bacterial homing capabilities and established use as a therapeutic chassis, BEVs could potentially revolutionize the approach to overcoming bacterial antibiotic resistance. By delivering antibiotics directly to infection sites, BEVs enable high doses to be administered without causing systemic toxicities [142]. In the works of Weiwei Huang et al., they discovered that stress-growing *A. baumanni* in medium spiked with sub-Minimal Inhibitory Concentrations (MIC) of levofloxacin elevated the production of BEVs which contains high levels of levofloxacin (Fig. 6C) [143]. This was deduced to be a form of drug efflux mechanism for antibiotic resistance. This phenomenon was utilized to generate levofloxacin-enriched BEVs, safeguarding the cargo from harsh environmental conditions. More importantly, levofloxacin-enriched BEVs exhibited superior bactericidal effects compared to equivalent amounts of free levofloxacin in a mouse intestinal Enterotoxigenic *Escherichia coli* (ETEC) infection model. Additionally, treatment with levofloxacin-enriched BEVs resulted in reduced adverse effects. This example illustrates how BEVs can contribute to combating antibiotic-resistant bacteria by delivering high doses of antibiotics while minimizing unwanted side effects. In addition to delivering high doses of antibiotics to combat bacterial antibiotic resistance, repurposing currently available antimicrobials is also a viable strategy to address antibiotic resistance [144]. One such example is rifampicin (Rif). Rif is an antibiotic traditionally employed to treat Gram-positive *S. aureus* infections [144]. However, it is ineffective against Gram-negative bacteria due to its inability to permeate through the double membrane structure of Gram-negative bacteria [145]. This deadlock is broken in the research conducted by Shuang Wu et al., in which Rif is loaded into mesoporous silica nanoparticles, which are then coated with BEVs derived from Gram-negative *E. Coli* (Fig. 6D) [146]. The coating of BEV conferred the biomimetic nanosystem with Gram Negative bacteria homing capabilities, resulting in preferential uptake by *E. Coli* even in the presence of S. aureus, and achieving unprecedented permeation of Rifampicin (Rif) into *E. Coli* bacterial cells. Furthermore, the antibacterial effects of the nanosystem were superior both in vitro and in an intraperitoneal infection mouse mode. In short, the same-type homing capabilities of BEVs have been utilized to deliver impermeable antibiotics to gram-negative bacteria.

Other than small molecules, BEVs can also carry biologics as therapeutic cargoes, which can protect them from degradation by proteases or physiological conditions during circulation to the target site [147]. This is embodied in the works of Wenjing Zai, in which the enzyme Catalase was loaded into *E. Coli* BEVs for hypoxia relief and enhancing radiotherapy of tumors [148] (Fig. 6E). The effectiveness of radiotherapy is greatly limited by hypoxic conditions in tumors, and this can be potentially reversed by the delivery of oxygen-evolving Catalase [149, 150]. Catalase-containing BEVs from hydrogen peroxide-stressed *E. Coli* cells were isolated and their catalytic activities were retained even under treatment with proteases. The BEVs not only effectively increased the oxygen levels in tumor cells and enhanced the effects of radiotherapy but also induced an immune response in CT26 tumor-bearing mice. Overall, the BEVs provided hypoxia relief in tumor cells, leading to subsequent synergistic radio and immune therapy. This study illustrates the capacity of BEVs to accommodate a diverse array of cargoes, including biologics, protecting them from protease degradation while also eliciting an immune response for synergistic treatments.

#### Functional agents for combinational therapy

Taking advantage of bacteria-derived extracellular vesicles, the delivery platform could not only prolong the blood circulation time of drugs but also accumulate therapeutic cargo in the targeting lesion region [129, 151]. In recent decades, with the witness of the remarkable development of nanotechnology and function material [106], the application of BEVs-based drug delivery was further expanded to integrate with multimodality therapy against diverse diseases (e.g. bacteria infection, cancer, etc.) by loading the nanomaterial with naturally-produced membrane vesicles. So far, the emerging advances in functional materials shine the light to explore biomedical applications, that could respond to external stimulation to produce cytotoxic substances (e.g. reactive oxygen species, heat, etc.) and achieve the therapeutic target [152–154]. The thriving development of nanosized function materials also boosts the establishment of novel strategies, like photodynamic (PDT) [155, 156] and photothermal therapy [157, 158], for the treatment of various health conditions, including tumor eradication and elimination of bacterial infections. However, these biomedical nanomaterials (e.g. gold nanoparticles, mesoporous silica nanoparticles, metal–organic frame, polymers, etc.) [159, 160] as external components always lead to off-target accumulation in healthy tissue, poor solubility in the physiological environment, and concerned biocompatibility. Thus, OMV has become an ideal cargo delivery platform to encapsulate the functional nanoagents to formulate “biomimetic nanoparticles” [161–164]. Inheriting the virtues of the OMVs, the delivery platform could active the immunotherapy within the lesion site by the naturally-presented adjuvants on the OMVs, while the complementary therapeutics loaded in the delivery platform boost the synergistic and enhanced therapeutic efficacy compared with parent components alone [165].

**Fig. 7:**
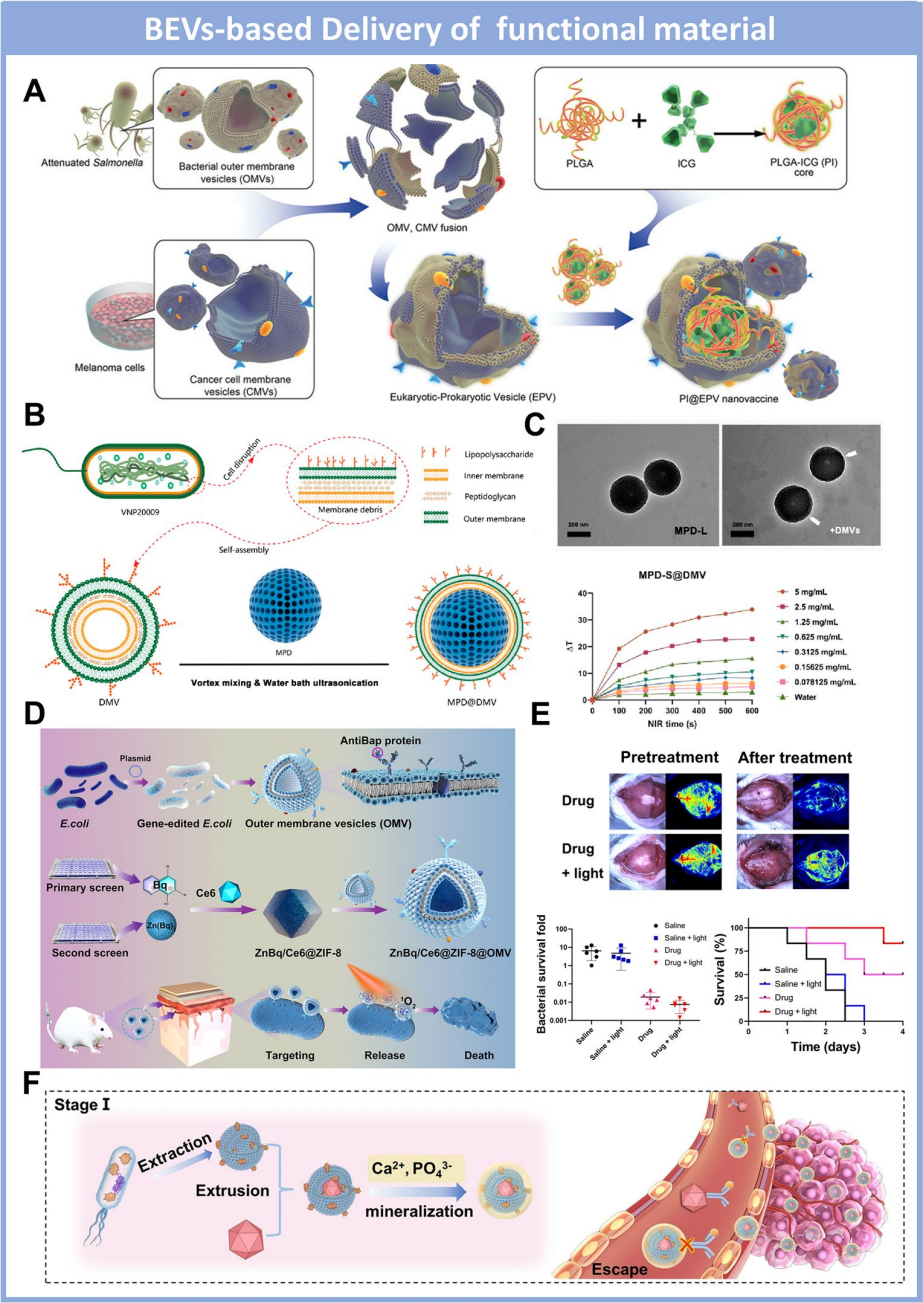
BEVs-based delivery platform loading functional materials for combinational therapy. **A**. Schematic illustration of Hybrid Eukaryotic–Prokaryotic Nanoplatform (PI@EPV) encapsulating the PLGA–indocyanine green to boost the synergistic antitumor effect. Reproduced with permission [166] Copyright 2020, John Wiley and Sons. **B**. Scheme of construction route for OMV-coated polydopamine nanoparticle (MOD@DMV) to augment antitumoral photothermal-immunotherapy; **C.** TEM images of MOD@DMV morphology and Characterization of the photothermal performance. Reproduced with permission [167]. Copyright 2023, American Chemical Society. **D**. Illustration of the targeted biomimetic delivery platform (ZnBq/Ce6@ZIF-8@OMV) to transport the metalloantibiotics for eradicating multidrug-resistant bacteria; **E**. In vivo antibacterial effect of ZnBq/Ce6@ZIF-8@OMV eliminating bacteria in the brain tissue and promoting the survival of infected mice. Reproduced with permission [168]. Copyright 2024, The American Association of the Advancement of Science. **F**. Schematic presentation of biomineralized *E. Coli*-derived outer membrane vesicles encapsulating the oncolytic adenoviruses to mediate the tumor cell autophagy for systematic immunotherapy. Reproduced with permission. [169] Copyright 2023, Springer Nature

Particularly, photothermal therapy was established as a powerful strategy to supplement the immunotherapy within the lesion site by OMV-based nanopharmaceuticals integrated with functional agents. Chen et al. have developed the Hybrid Eukaryotic–Prokaryotic Nanoplatform (PI@EPV) encapsulating the poly (lactic-co-glycolic acid)–indocyanine green (PLGA-ICG) to boost the synergistic antitumor effect (Fig. 7A) [166]. By fusing melanoma cytomembrane vesicles (CMVs) and *Salmonella*-derived outer membrane vesicles (OMVs), the PI@EPV nanoplatform could efficiently localize inside the tumor site, stimulating the antitumor immune response, including both the dendritic cell maturation and activation of cytotoxic T lymphocytes. Moreover, the localized photothermal agent ICG could efficiently transfer the near-infrared stimulation into hyperthermia to initiate the immunogenic cell death, subsequently producing tumor-associated antigens to augment the immunotherapy efficacy by PI@EPV. Similarly, different photothermal agents have been employed in the OMVs to construct integrated nanopharmaceuticals with improved cytotoxic immune response. Chen et al. have wrapped the outer membrane vesicles from *Salmonella VNP20009* onto the mesoporous polydopamine nanoparticles, which is the core component to mediate the photothermal response (Fig. 7B, C) [167]. The successful construction of integrated nanocomposite (MPD@DMV) led to passive localization in the tumor site and the synergistic tumor regression in the B16F10 melanoma mice model, attributing to the T cell infiltration and significant release of antitumoral cytokines. Notably, the intravenous injection of MPD@DMV could activate better long-term immune response compared with intertumoral administration, potentiating further clinical translation for tumor vaccination.

Moreover, the drug delivery platform based on OMVs could also encapsulate the functional material for combinational therapy against the bacteria infection, benefiting from OMV’s bacterial-specific targeting ability. Recently, Wei et al. have developed the targeted biomimetic delivery platform to transport the metalloantibiotics for eradicating multidrug-resistant bacteria (e.g. *A. baumannii*) [168]. The OMV derived from *E. Coli* was genetically modified to anchor the targeting antibody fragment, realizing the specific recognition of *A. baumannii*. After the systematic screening, the metal complex Zn(Bq)_2_ was selected as the efficient killing component loaded in the metal–organic frame (zeolitic imidazolate framework-8) to maintain stability and enhance the loading efficiency in the OMV-based delivery platform. Importantly, the photosensitizer (Chlorine6) was co-loaded into the delivery platform to construct the final nanomedicine named ZnBq/Ce6@ZIF-8@OMV, suggesting the synergistic bacteria eradication by intense ROS production during the photodynamic treatment (Fig. 7D). Such an OMV-based delivery platform shows great potential for disruption of *A. baumannii*–infected biofilm and acceleration of mice meningitis recovery (Fig. 7E).

Notably, the integration with functional material and OMV-based cargo delivery platform not only enhances the therapeutic performance by combinational modality but also promotes the stability and biosafety of nanopharmaceuticals in complicated physiological environments. During the circulation of OMV in the blood, it could build up complicated interactions with neutrophils, endothelial cells, and other immune-related cells to stimulate the systematic inflammatory response. However, the overreacted immune response also becomes a potential concern of OMV to appropriate application in the clinical trial. To maintain the sufficient immunogenicity and intact compound of OMV, the biomineralization by abiotic material on the OMV surface could be the proper strategy to enhance the bioavailability and therapeutic efficiency of the BEVs-based drug delivery system [170]. In particular, Chen et al. genetically modified *E. Coli* to harvest the melanin-rich OMVs for targeted photothermal immunotherapy in the tumor region [171]. More importantly, to improve the systemic biosafety of OMVs, the nanopharmaceuticals were further functionalized by the calcium phosphate as the outer layer, alleviating the overreacted inflammatory response and liver damage with intravenous administration. The biomineralized OMVs exhibited an outstanding antitumor immune response combined with photothermal efficacy, potentiating the effective suppression of tumor progression and recurrence. Similarly, Ban et al. have developed engineered *E. Coli*-derived outer membrane vesicles encapsulating the oncolytic adenoviruses to mediate the tumor cell autophagy for systematic immunotherapy (Fig. 7F) [169]. The OMVs were genetically engineered to express the pyranose oxidase on the surface and further modified with the biomineralization of calcium phosphate to prevent elimination by the innate immune system and promote the biosafety of nanocomposites. After internalization in the tumor cells, the pyranose oxidase could boost the production of H_2_O_2_ to initialize the tumor autophagy and autophagosome formation, subsequently promoting viral replication and finally leading to cell death. Such OMV-based nanocomposite illustrated the cascade-amplified immunotherapy and successful attenuation of TC-1-hCD46 xenograft tumor growth.

### BEVs as nanovaccines

Vaccines are therapeutic formulations designed to elicit a host immune response, enabling the host organism to develop long-term immunity against the actual pathogen in the future [172]. Vaccines should engage with immune cells of the innate response, typically characterized by the secretion of pro-inflammatory cytokines and recruitment of neutrophils or macrophages [173, 174]. To ensure long-term immunity and protection against the intended pathogen, vaccines must also trigger the adaptive immune response, usually hallmarked by the activation or maturation of APCs, T cells, and B cells, and the secretion of antigen-specific antibodies [175]. Some current vaccines developed are based on attenuated or inactivated pathogens such as bacteria or viruses [172], which carry PAMPS and antigens to activate the immune without being virulent. However, their safety remains a topic of debate [176]. As derivatives of bacteria, BEVs also contain PAMPS which allows them to interact with immune cells just like whole bacterial cells, suggesting their potential role as vaccines themselves or as adjuvants to assist in boosting immune responses. Their non-propagative nature is considered safer than using live bacterial vaccines [176] and with recombinant technology, BEVs can be engineered to modulate their immunogenicity or even express foreign antigens for cross-species immunity. The feasibility of BEVs as vaccination platforms is demonstrated by the limited success of a pioneering *Neisseria meningitidis* BEV-based vaccine [177]. This has inspired many recent advances in BEVs-based nanovaccines against a wide range of pathogens and diseases.

#### Bacterial infections

**Fig. 8:**
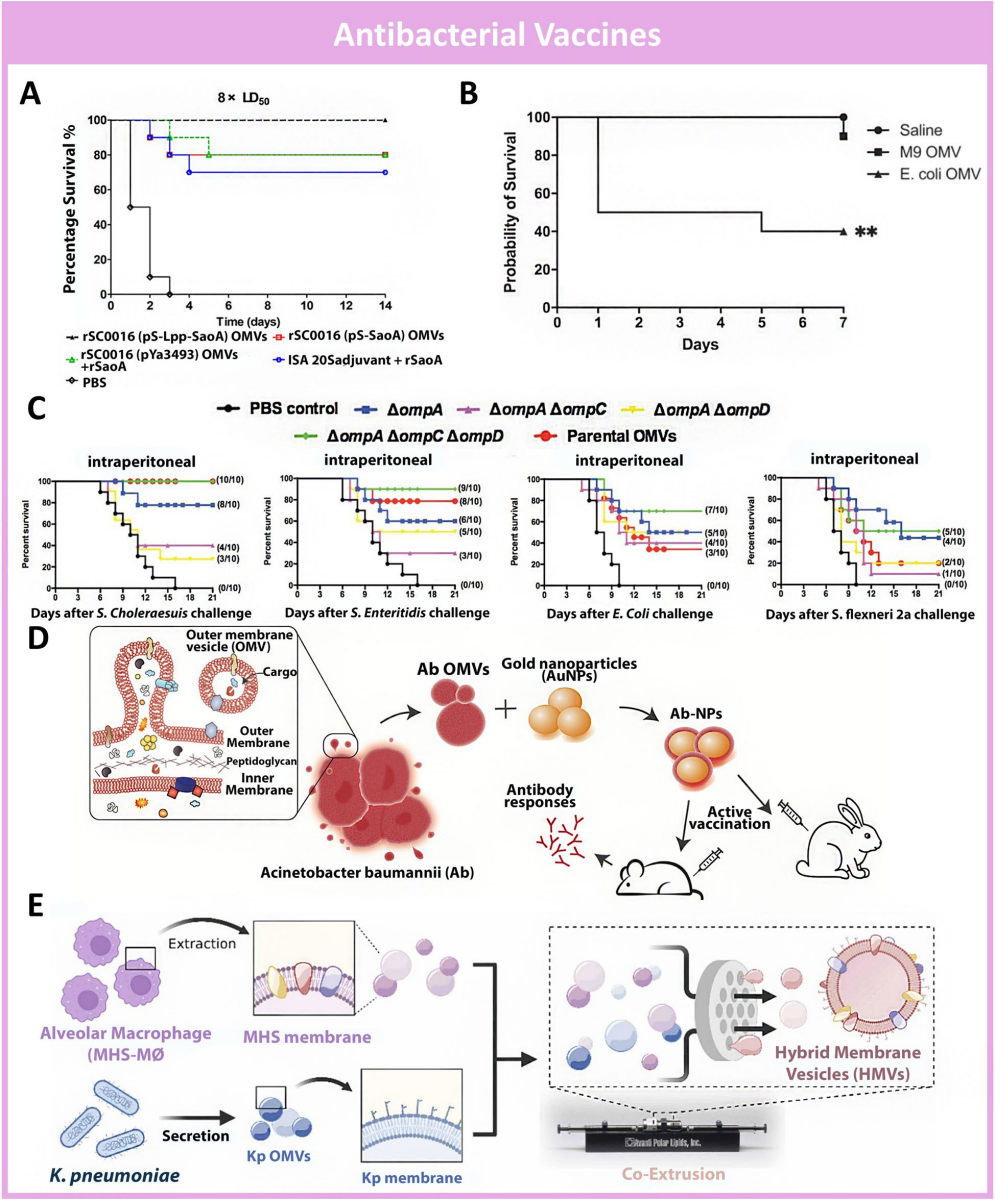
Schematic of BEVs based nanovaccine against bacterial infections. **A.** BEV nanovaccine with SaoA-Lpp protected mice against *S. Suis* infections. Reproduced with permission. [178] Copyright 2023, American Society for Microbiology **B.** BEV-based vaccine enriched with T3SS-3 and T6SS-1 (M9-OMV) protected mice against virulent *B. pseudomallei*. Reproduced with permission [181]. Copyright 2021, Springer **C.** *ΔompAΔompCΔompD* BEVs nanovaccines protected mice against all *Salmonella strains and* APEC O78 challenges. Reproduced with permission [185]. Copyright 2020, Frontiers **D.** Schematic of Hybridization of BEVs with Au nanoparticles results in higher homogeneity in size and increased immune response. Reproduced with permission. [190] Copyright 2022, Wiley–VCH. **E.** Schematic of hybridization of BEVs with macrophage membranes for increased biocompatibility and modulated immune response. Reproduced with permission. [192] Copyright 2024, Elsevier Inc

Benefited by their ease of engineering, BEVs are allowed to express non-native proteins or protein conjugates in high numbers via recombinant technologies [21]. This concept was effectively implemented in the research conducted by Li et al., where engineered BEVs of *Salmonella enterica* expressing surface lipoprotein-SaoA were developed as a vaccine against *Streptococcus suis* infection (Fig. 8A) [178]. SaoA is a surface-anchored protein that is highly conserved in different *S. suis* species which has great potential to be developed as a vaccine antigen. SaoA was conjugated to the C terminus of surface membrane Lipoproteins (Lpp) and expressed on the surface of BEVs of *S. suis* for optimal immunogenicity. Mice injected with BEVs with SaoA-Lpp conjugates have higher levels of cytokines, anti-SaoA antibodies in serum, and lower bacterial counts in their blood and brain tissues, as compared to other control groups. When subjected to a 50% lethal dose (LD50) of S. suis serotype 2, 100% of mice receiving BEVs with SaoA-Lpp conjugates survived whereas PBS-treated mice died within 3 days. This study demonstrates the potential of BEVs being able to be engineered such that inaccessible proteins can be recombinantly expressed on the surface of BEVs to maximize their interaction with host immunity cells and improve antigen-specific antibody responses [179].

The development of BEV-based vaccines often involves genetic engineering and altering the expression of PAMPS found on the outer surface to modulate their immunogenicity or to incorporate foreign antigens for cross-immunity. This is an often expensive and tedious process [180]. The alteration of PAMPS on bacteria and BEVs can also be achieved by cultivating them in modified mediums. For example, Baker et al. demonstrated that *Burkholderia. Pseudomallei* cultivated in a medium deprived of iron and zinc would express virulent factors Type three secretion system (T3SS-3) and type six secretion system, (T6SS-1) [181]. These virulent proteins allow *B. Pseudomallei* to reside in macrophages and survive in hosts [182]. Based on the hypothesis that BEV vaccines expressing these virulent factors would induce a more effective immune response, Sarah et al. developed a *B. pseudomallei* BEV-based vaccine that was enriched with T3SS-3 and T6SS-1 (M9-OMV) (Fig. 8B). Although M9-OMV was enriched with virulent factors, they exhibited no toxicity to living cells and protected mice against virulent *B. pseudomallei* alongside with live attenuated *B. pseudomallei* vaccines (Bp-82). M9-OMVs outperformed Bp-82 in terms of eliciting immune responses by inducing higher levels of IgG in serum, and actively engaging with T cell and dendritic cells. This study promises an effective BEV-based vaccine against *B. Pseudomallei* which outperforms live attenuated bacteria vaccines, without possessing the inherent safety risks of replicating live vaccines. Furthermore, the engineering of such a vaccine is achieved simply by modifying the cultivation conditions of the parent bacteria without genetic engineering.

The development of successful antibacterial vaccines is often difficult due to the multiple serotypes of these pathogenic bacteria [183]. A broad-spectrum vaccine offering protection against most pathogenic serotypes is highly demanded [184]. By truncating the Outer Membrane Proteins (OMPs) of Salmonella Typhimurium χ3761 and their BEVs, Yuxuan Chen et al. managed to develop a broad-spectrum vaccine that provided cross-protection against various *Salmonella* and Avian Pathogenic *Escherichia coli* O78 (APEC O78) in mice and chickens [185] (Fig. 8C). The study was based on the theory that the deletion of major OMPs in BEVs may affect the expression of conserved OMPs and therefore affect the cross-protection of such BEV vaccines. In mice, the Δ*ompC*Δ*ompD Salmonella Typhimurium* UK-1 BEVs induced higher IgG and IgA levels, but immunization with *ΔompAΔompCΔompD* BEVs protected mice against all *Salmonella strains, Shingella and* APEC O78 challenges. Similarly in chicken models, *ΔompAΔompCΔompD* immunized chickens were found to survive *S. Enteritidis* and APEC O78. These suggest that *ΔompAΔompCΔompD* BEVs could be a feasible broad-spectrum vaccine against Colibacillosis-causing bacteria tested in the study. In another example, a well-established *Yersinia pseudotuberculosis-based* nanovaccine platform was genetically engineered to highly express PspA and developed into a broad-spectrum vaccine (OMV-PspA) against influenza-mediated secondary *Streptococcus pneumoniae* (Spn) infections [186]. OMV-PspA was able to induce the highest levels of anti-PspA IgG2a/IgG1 and IgG2b/IgG1 than control groups and inducing T cell responses. The OMV-PspA also protected mice against Spn D39 and Spn A66.1 challenges after initial CA04 (H1N1) challenges after 205 days post-vaccination. Through the engineering of BEVs, broad-spectrum vaccines that provide cross-species immunity can be developed owing to the similarities in the expressions of PAMPs across different bacterial species and their BEVs.

Previous attempts to develop BEV-based vaccines have faced challenges due to the heterogenicity in size, composition, and internal cargoes of extracted BEVs [187]. On the other hand, nanoparticles such as citrate-stabilized gold nanoparticles (AuNPs), have very narrow size distributions and are consistent in their size [188]. Furthermore, AuNPs have been reported to have superior affinity to immune cells, making them suitable carrier candidates for vaccine development [189]. In the works of Elisabet Bjanes et al., AuNPs were coated with the BEVs of *A. baumannii* to develop a hybrid nanovaccine against *A. baumannii* pneumonia and sepsis [190] (Fig. 8D). The hybridized nanovaccine (Ab-NP) exhibited a high degree of uniformity in size, unlike the crude BEVs extracted from A. baumannii. It induced higher levels of immunoglobulin G (IgG), increased percentage of B cells, and expression of activation markers in dendritic cells. This indicated that the homogeneity of AB-NPs compared to Ab-OMV contributed to the enhancement of Antigen Presenting Cells. Ab-NPs and postvaccination serums protected rabbits for up to 6 months in sepsis infection and intratracheal pneumonia models. This study demonstrated the compatibility of BEVs with nanoparticles and the importance of particle size homogeneity in the performance of BEV-based vaccines. It has been well-reported that BEVs participate in the pathogenicity of bacterial invasion by transmitting virulent factors such as LPS, which can result in inflammatory responses [191]. Thus, it is possible that BEV-based vaccine platforms can cause over-immune stimulation, hyperinflammation, or damage to host tissues. In order to mitigate these shortcomings of BEV-based vaccines, BEVs of *Klebsiella pneumoniae* were hybridized with alveolar macrophage membrane to form an intratracheal vaccine (HMV) [192] (Fig. 8E). When administered to mice, pure BEVs damaged lung epithelial cells while HMVs did not affect the growth of epithelial cells and induced lower levels of cytokines as compared to BEVs. Mice immunized with HMV had high levels of IgM and IgA in serum and survived subsequent *K. pneumoniae* challenges. This study demonstrates the versatility of BEVs to be hybridized with mammalian membranes to increase their biocompatibility and modulate their immunogenicity.

#### Viral infection

**Fig. 9:**
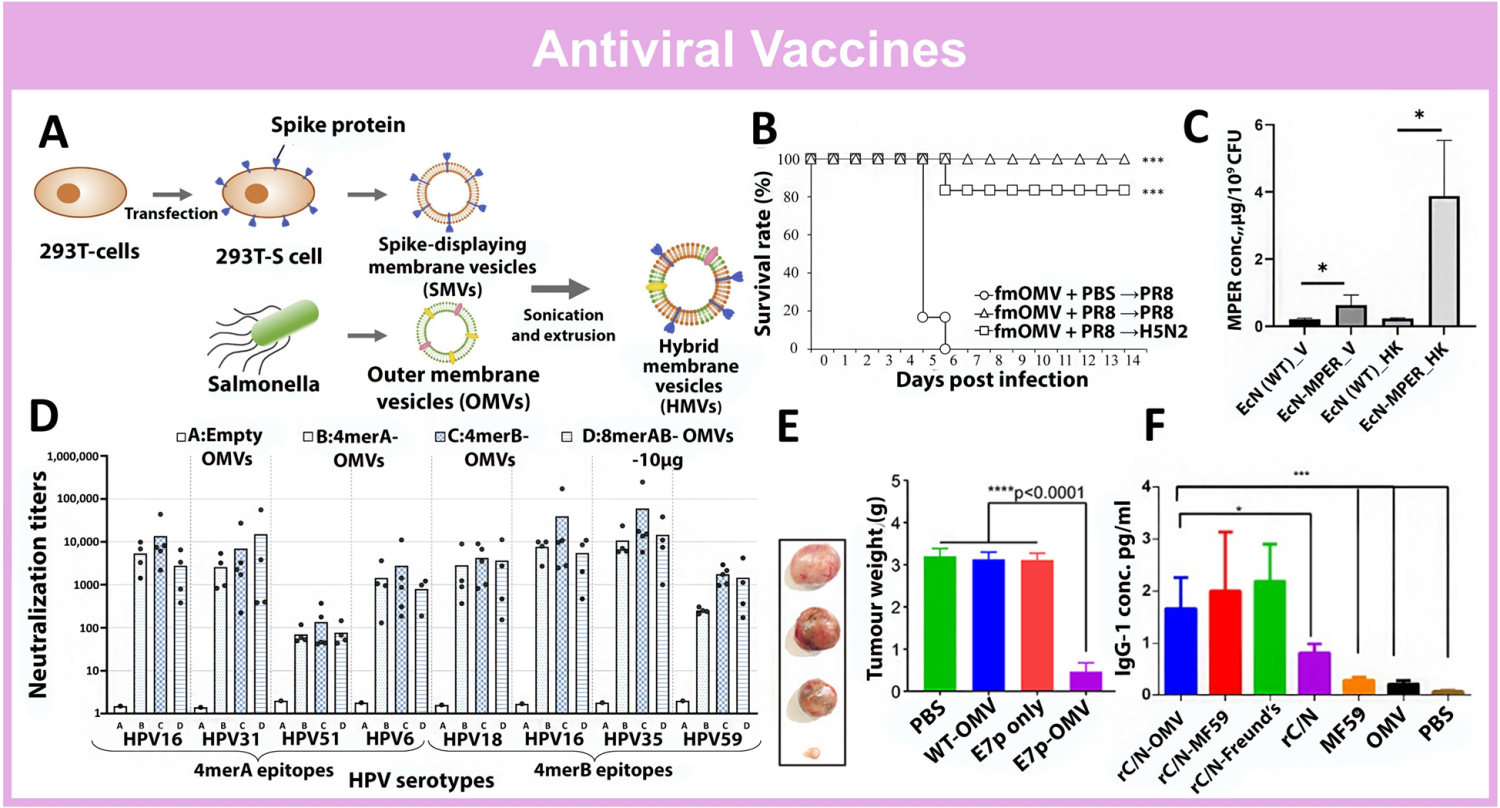
BEV-based nanovaccines against viral infections. **A** Schematic of SARS-CoV-2 spike protein displaying virus-mimetic hybrid membrane-derived vesicles (HMVs) against SARS-CoV-2. Reproduced with permission. [198] Copyright 2022, Elsevier Inc. **B** LPS modified on BEVs protect mice against different serotypes of Influenza viral infection. Reproduced with permission [205]. Copyright 2022, Elsevier Inc. **C** Incorporation of HIV-1 envelope membrane-proximal external region (MPER) with outer membrane protein OmpF on BEVs nanovaccines induces production of anti-HIV 1 antibodies. Reproduced with permission [214]. Copyright 2024, Springer Elsevier Inc **D** L2 protein polytope displaying BEV-based vaccine induces L2-Specific IgG Titers against 8 serotypes of HPV. Reproduced with permission [216]. Copyright 2023, MDPI. **E** HPV E7 loaded BEV nanovaccine exhibits anti-tumour efficacies. **F** BEV nanovaccine expressing fusion HCV proteins induces IgG-1 in mice. Reproduced with permission. [220] Copyright 2019, Iranian Biomedical Journal

The COVID-19 outbreak has prompted urgent efforts to develop a vaccine against SARS-CoV-2[193]. While mRNA vaccines like Moderna's have seen widespread administration [194], they are hampered by challenges related to instability and transfection efficiency [195, 196]. BEVs can express and stabilize recombinant antigens and their innate immunogenic properties allow them to act as adjuvants to enhance immune responses [197]. With the intention of developing a BEV-based vaccine against SARS-CoV-2, Liu et al. hybridized SARS-CoV-2 spike protein displaying cell membrane vesicles with the BEVs of *Salmonella typhimurium* to form virus-mimetic hybrid membrane-derived vesicles (HMVs) (Fig. 9A) [198]. The spike proteins are characteristic of SARS-CoV-2 and are involved in the pathogenicity of the virus [199], qualifying them as ideal antigens for vaccine development. The spike proteins were initially recombinantly expressed on mammalian cells to preserve their native three-dimensional structure [200], while BEV components serve as adjuvant to boost the immune response. The HMVs were quickly internalized by DCs and increased MHC expression. Mice immunized with HMV exhibited a biased Th2-mediated humoral response and induced active T cell response with an increased expression of IFN-g and IL-6. This suggests that HMVs could serve as potential candidates for a BEV-based vaccine against SARS-CoV-2. Successful vaccines are intended for intramuscular administration and inducing systemic immunity. However, intranasal vaccines can induce local mucosal immunity and prevent further transmission of diseases [201]. Consequently, an intranasal vaccination strategy based on *Neisseria meningitidis* BEVs carrying D614G spike protein (mC-Spike) of SARS-CoV-2 was developed [201]. The nanovaccine (OMV-mC-Spike) was composed of LPS-bound HexaPro Spike-mCRAMP conjugate, in which mCRAMP contributed to binding to LPS on the BEV surface while HexaPro Spike [202] served as a previously optimized antigen of SARS-CoV-2. Intranasal administration of OMV-mC-Spike to mice induced higher levels of IgG and IgA titers in serum, nasal washes, and lungs. Hamster models subjected to the viral challenge after immunizing with OMV-mC-Spike displayed the lowest viral loads and reduced lower respiratory tract diseases afterward. The versatility of BEVs to accommodate different cargoes and antigens allows the rapid development of BEV-based vaccines against novel diseases.

The influenza virus is an RNA-based virus, and its frequent mutations lead to changes in antigenicity, complicating the development of a cross-subtype vaccine [202, 203]. Doo-Jin Kim’s group has previously developed BEV-based vaccines using *E. Coli* BEVs, wherein the LPS is modified with a lipid A 40-phosphatase (fmOMV) [204]. The adaptive immune response triggered by fmOMV was investigated recently (Fig. 9B) [205]. Mice injected with fmOMVs exhibited high levels of HA-specific antibodies, as well as HA- and NP-specific IgA and IgG antibodies, which suggest a cross-immunity against influenza mutants. When challenged with several strains of influenza, fmOMV immunized mice displayed pronounced IFN- γ -producing T cells and were protected against all tested strains 18 weeks post-vaccination. This study demonstrates the immunogenicity of modified BEVs alone, without viral antigen can be utilized as vaccines against influenza.

Human-immunodeficiency virus (HIV), Human Papilloma Virus (HPV), and Hepatitis C (HCV) are sexually transmitted viruses that cause diseases such as AIDS, Cervical Cancer, and Liver Cancer [206]. These diseases are chronic or lifelong, posing a significant global health challenge that requires urgent attention and solutions [207]. Furthermore, there are currently only medications available to manage the symptoms of these diseases, with no cure currently available [208]. Vaccination is the only effective method to prevent the spread of these diseases but so far, only approved vaccinations against HPV are available [208, 209]. As highly immunogenic carriers and versatile platforms for the expression of foreign proteins, BEVs may be used for the development of antiviral vaccines as they can act as adjuvants or express viral antigens via recombinant technology. Earlier attempts at developing BEV-based vaccines against these viruses and diseases have been made [210, 211].

The quest for an effective HIV vaccine has been an arduous journey spanning over 40 years [212]. Prototypes developed thus far often suffered from adverse effects and exhibited weak targeting capabilities [213]. A recent endeavor to develop a BEV-based HIV vaccine involved incorporating the HIV-1 envelope membrane-proximal external region (MPER) into the outer membrane protein OmpF as a construct in *E. Coli* Nissle 1917 and their BEVs (Fig. 9C) [214]. The MPER-OmpF decorated BEVs were antigenic to MPER-binding HIV-1 gp41 (2F5) monoclonal antibody, suggesting its potential as a viable vaccine candidate awaiting further studies.

Human papilloma viruses (HPV) are non-enveloped viruses consisting of more than 200 subtypes, 15 high-risk subtypes known to cause Cervical Cancer [215]. Current clinically applied HPV vaccines are based on virus-like particles composed of the L1 protein. However, they are limited by their type restriction, instability, and high production costs [216]. Prior studies using HPV L2 proteins provided wide strain protection as the L2 protein sequence contains a major cross-neutralization epitope and induced broadly neutralizing anti-HPV antibodies [216, 217]. A recent advance in HPV vaccine development involved the construction of a BEV-based vaccine displaying an L2 protein polytope that is made up of amino acid sequences from 8 HPV serotypes (Fig. 9D) [216]. The *E. Coli* BL21(DE3)∆60 BEV-based vaccine induced L2-Specific IgG Titers in immunized mice and neutralizing titers in in vitro pseudovirus neutralization assay. A laboratory-scale production process was also conducted to demonstrate the scalability of the production of this BEV-based vaccine. The limitation of current HPV vaccines is that they only protect against HPV naïve individuals and provide no therapeutic value to HPV-positive or patients with cervical cancer. A tentative therapeutic vaccine was prototyped by Chen et al., in which the HPV-associated peptide E7 was recombinantly expressed in *E. Coli* BEVs to form a nanovaccine (Fig. 9E) [216]. E7p-OMV protected the internally loaded E7 peptides from proteases and when injected into mice, displayed tumor suppressive effects. This was found to be mediated by increased antigen expression in DCs, specific Th1 (CD4 + IFN-γ +) responses, and CTL (CD8 + IFN-γ +) responses. This study has the potential to pave the way for future developments in therapies for HPV-associated cancers, which are currently lacking and in high demand.

Cirrhosis and hepatocellular carcinoma are caused by Hepatitis C (HCV), and currently, there are no clinically licensed vaccines available for prevention against HCV infections [218]. Prior attempts to develop an HCV vaccine involved the truncation of protein NS3 but were faced with a weak invocation of the immune response [219]. An adjuvant may be required to boost the immune responses of such vaccines. A recent study developed a *Neisseria meningitidis* serogroup B BEV-based vaccine platform with recombinant truncated core1-118 (rCore) and NS31095-1384 (rNS3) fusion protein (rC/N) against HCV (Fig. 9F) [220]. The rC/N-BEV vaccine induced higher IgG1 and IgG2a levels, indicating a Th1/cellular response and outperforms combinations of rC/N with other adjuvants. This study demonstrates the superior adjuvant properties of BEVs and their potential as a platform for the development of future HCV vaccines.

#### Cancer

Notably in recent decades, the development of vaccines has been recognized as a new weapon to combat tumors and potentiate the expansion of oncologic armamentarium [221]. Among the emerging nanovaccines and pharmaceuticals, bacteria and their derived extracellular vesicles attract great interest to be applied as the new generation of cancer vaccines to achieve efficient immunotherapy [26, 222, 223]. Particularly, bacteria localized in the tumor microenvironment could build up the precise interaction between cancer cells, tumor-infiltrating immune cells, and other overexpressed biomarkers (e.g. cytokines, chemokines), remodeling the immune-suppressed microenvironment in the tumor site. The abundance of pathogen-associated molecular patterns in the bacteria demonstrates the desirable immunogenicity to activate the systemic immune response [224]. However, injection of intact bacteria containing intracellular contents (e.g., endotoxins, genetic material, etc.) into the patients always leads to severe safety concerns and potential side effects, thus the improvement of bacterial-inspired vaccines is highly demanded [225, 226]. The bacterial extracellular vesicles, especially outer membrane vesicles (OMV), show great promise to maintain the immunogenicity for in situ immune activation with considerable biosafety compared with weakened bacteria. Moreover, the small size of OMV (20–250 nm) also benefits the lymphatic drainage and long-term accumulation of antigens by enhanced permeability and retention (EPR) effect, thus improving the immunotherapy specificity [227, 228]. Particularly, Kim et al. first reported that using *E. Coli* OMV as cancer immunotherapeutics, which could efficiently localize in tumors due to their nano-size, inducing the production of interferon-γ and CXCL10 cytokines for tumor regression [227]. Besides, thanks to the hollow structure of OMVs for encapsulating the immunoadjuvant or antigens payload into the BEVs, the bacterial-derived nanopharmaceuticals could further reshape the suppressed immune environment in situ as antigen sources and boost the therapeutic efficiency, potentiating their great promise as a novel nanoplatform for the cancer vaccine’s development.

Benefiting from the advanced gene engineering technologies and molecular biological methods (e.g. DNA transfection, CRISPR-cas9, Gene sequencing) [229], diverse engineered bacteria have been developed to originally produce the antigens or cytotoxic protein compounds, flexibly modify the payload in the extracted BEVs or functionalize the nanopharmaceuticals for versatile tumor vaccination. For instance, to directly deliver the therapeutic cargo to the tumor site, Chiang et al. have genetically tailored probiotics *E. Coli* Nissle 1917 to secret the therapeutic OMVs loaded with small cytotoxic protein (HlyE) [230]. Oral administration of engineered bacteria illustrated effective tumor colonization. In response to the arabinose metabolism, HlyE-loaded OMVs contained personalized antigens to boost the dendric cell uptake, suggesting the significant tumor regression in the xenograft colorectal tumor model.

**Fig. 10:**
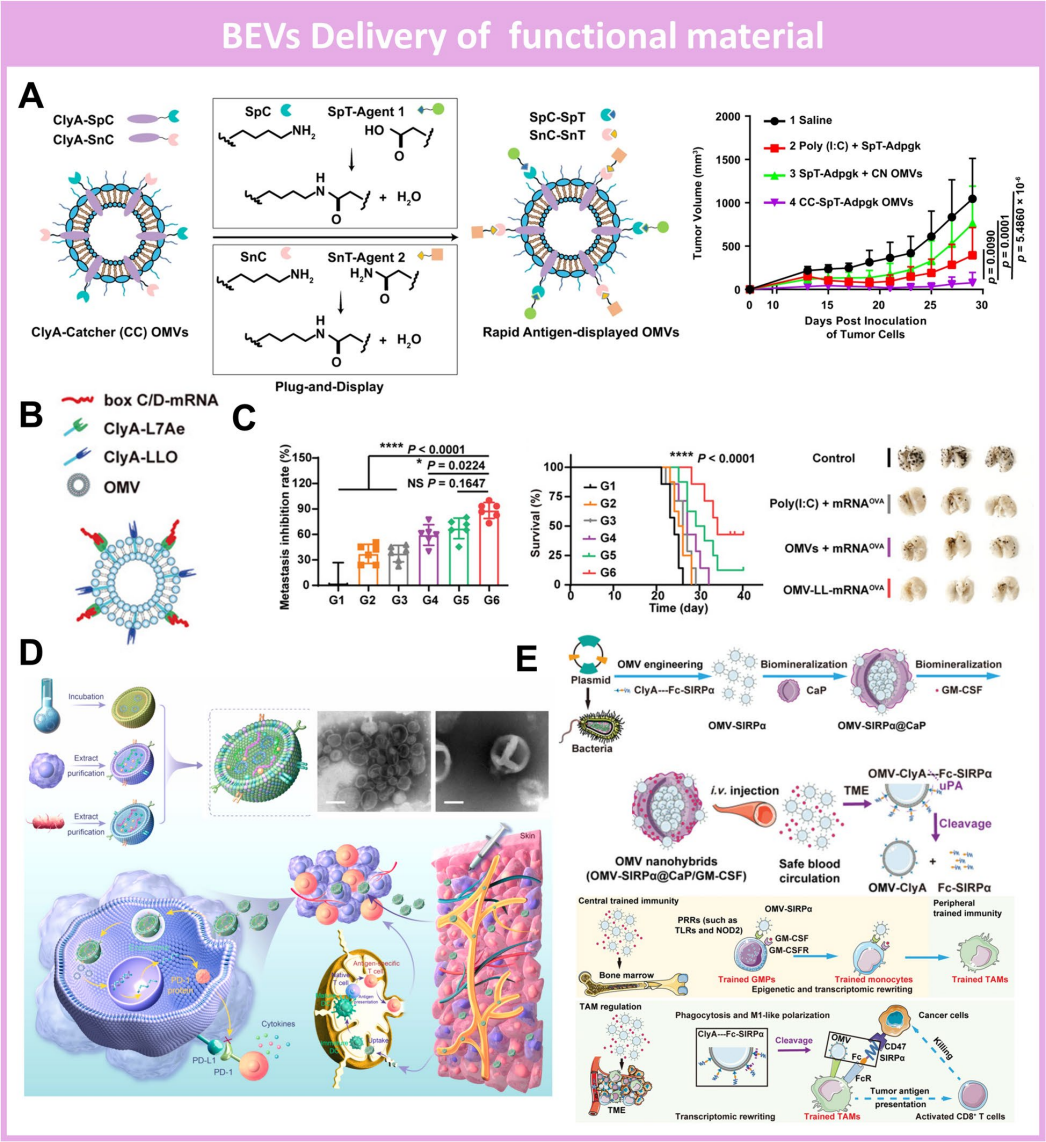
**A**. Schematic illustration of OMV-based vaccine platform by fusing the diverse protein catchers (Spycatcher and Snoopcatcher) on ClyA for versatile antigen display, suggesting promising antitumor immunity in the MC38 tumor model. Reproduced with permission [231] Copyright 2021, Springer Nature. **B**. Scheme of OMV-based mRNA delivery system by fusing the RNA-binding protein L7Ae and endosomal escape-promoting protein LLO on OMVs surface; **C**. In vivo antitumor efficacy and long-term immune memory for metastasis inhibition by OMV-based mRNA delivery system. Reproduced with permission. [232] Copyright 2022, John Wiley and Sons. **D**. Schematic presentation of fabricated Lipo@HEV by membrane fusion for synergistic cancer immunotherapy, supplemented by targeting delivery of PD-L1 trap plasmid. Reproduced with permission. [236] Copyright 2023, Elsevier. **E**. Schematic overview of OMV vaccine (OMV-SIRPα@CaP/GM-CSF) formation and biomineralization to enhance safe circulation in the blood, stimulating the trained immunity for distinct tumor regression. Reproduced with permission. [239] Copyright 2023, John Wiley and Sons

Moreover, genetic modification of bacteria could be leveraged to fuse the functional protein onto the cytolysin A (ClyA), which naturally existed on the membrane of BEVs, thus directly expressing antigens on the vesicle’s surface. The fused protein (e.g. enzyme, antibody, RNA-binding proteins, fluorescent protein, etc.) on the membrane could also endow the diverse function to enhance the therapeutic performance of BEVs-based cancer vaccines. Particularly, Cheng et al. have developed a versatile OMV-based vaccine platform by fusing the diverse protein catchers onto ClyA, which allows the OMV vaccine to present multiple and distinct tumor antigens on the surface (Fig. 10A) [231]. Using genetic engineering, the flexible OMV vaccine could fuse the target antigens onto the ClyA and activate the T cell for in situ antitumoral immune response. Importantly, the OMV vaccine has applied a Plug-and-Display system comprising diverse tag & catcher protein pairs, including the SpyTag (SpT)/SpyCatcher (SpC) pair and the SnoopTag (SnT)/SnoopCatcher (SnC) pair by fusing to the ClyA. By employing Plug-and-Display fused protein, different antigens could rapidly and simultaneously bind to OMV, realizing synergistic antitumor immunity, while the nanovaccine could efficiently abrogate lung melanoma metastasis and suppress colorectal tumor growth. Similarly, Li et al. have improved the “Plug-and-Display” strategy to deliver the mRNA antigens as the vaccine for antitumor immunotherapy (Fig. 10B) [232]. The RNA-binding protein, L7Ae, was fused onto ClyA and matched the binding sequence, box C/D was inserted into mRNA cargo for efficient loading of antigen on the OMV surface. Besides, the lysosomal escape protein, listeriolysin O was also fused with ClyA to improve the mRNA delivery efficiency in the dendritic cells (DCs), while the fused protein endowing the escape of nanovaccine from lysosome and cargo accumulation in the cytoplasm. The OMV-based mRNA vaccine could activate the innate immunity and subsequent cross-presentation in DCs, suggesting the efficient regression of melanoma and MC38 colon cancer model (Fig. 10C). Moreover, the genetic fusion on ClyA could also present the targeting ligand to improve the precise tumor targeting of OMV-based vaccines. Adriani et al. have genetically engineered the high-affinity anti-EGFR ligand, the single-chain variable fragments originated from panitumumab antibody, on the ClyA protein to construct the bioengineered OMVs for specific tumor accumulation, potentiating the application of these immunotherapy agents in different types of tumors [233].

To further improve the antitumor specificity of BEV-based vaccines, antigen-expressed cancer cell membrane particles, and extracellular vesicles have been employed to fuse with bacteria-derived vaccines to form hybridized nanopharmaceuticals [234]. Typically, the pure cytoplasmic membrane extracted from bacteria reserved abundant PAMPs to mobilize the immune system. L Chen et al. have hybridized the *E. Coli* cytoplasmic membrane and autologous tumor membrane to construct the fused membrane platform (HM-NPs), realizing the colocalization of antigens and adjuvants in the dendritic cell [235]. The bacterial cytoplasmic membrane was pretreated with lysozyme to remove the LPS and other cell wall components to prevent potential cytotoxicity. Such hybridized nanovaccine illustrates significant antitumor immune activation and diverse tumor ablation in colon, breast, and melanoma cancer mouse models. Benefiting from the fusion approach of BEVs and tumor EVs, recently, Tong et al. employed the outer membrane vesicle from *Akkermansia muciniphila* (Akk-OMV) as the self-immunoadjuvant to hybridize with antigen-rich tumor-derived exosomes for cancer vaccine construction (Fig. 10D) [236]. Besides, the Lipo@HEV formulation is supplemented with PD-L1 trap plasmid cargo to facilitate gene therapy for immune checkpoint inhibition, synergistically combating tumor growth by the hybridized OMV-based vaccine.

Different from conventional tumor vaccines inducing an adaptive immune response, recent advances in OMV-based vaccines try to improve immunogenicity by interfering with the immune-related signaling pathway. For instance, the long-term innate immune memory, known as trained immunity, which is referred to the procedure of modifying innate immune cells along with their hematopoietic progenitors to enhance nonspecific innate immunity [237, 238], particularly in reaction to subsequent infection or vaccination. The OMV-based vaccine could facilitate the trained immunity-related signaling pathway by the presence of PAMPs and other immunoadjuvants, which are independent of specific tumor-specific antigens. Particularly, Liang et al. have developed OMV-based nanohybrids by fusion of the recombinant protein of SIRPα and Fc fragment (SIRPα-Fc) on the surface to block the CD47-mediated immune escape (Fig. 10E) [239]. Following the subsequent calcium phosphate mineralization and loading of granulocyte–macrophage colony-stimulating factor (GM-CSF), the final OMV vaccine (OMV-SIRPα@CaP/GM-CSF) targets tumor-associated macrophages (TAMs) in the bone marrow to train the progenitor cells and monocytes for long term immunity. OMV-SIRPα@CaP/GM-CSF also illustrated efficient tumor regression by trained immunity in both MC38 tumor and B16-F10 tumor models with distinct T-cell-mediated immune responses, suggesting the different therapeutic mechanisms for vaccination formulation. Recent developments of BEV-based vaccination to enhance immunoreactivity were also considered to facilitate the cGAS-STING pathway in the dendritic cell to heighten the DC maturation. Zhang et al. constructed the interfacial nanocloak on the *B. fragilis*-derived extracellular vesicles by coating the biocompatible manganese oxide [240]. Once the nanocloaked BEVs internalized into the dendritic cell, the nanocloak layer will dissolve in the lysosome and release the Mn^2+^ to mobilize the cGAS-STING pathway for boosted maturation of DC, illustrating the amplified immunotherapeutic ability in breast cancer model.

## Advantages of BEVs

Extracellular vesicles are key mediators of intercellular communication, shuttling biological and chemical messengers throughout the host. Extracellular Vesicles are produced by bacteria (BEVs) and mammalian cells as well, known as mammalian extracellular vesicles (MEVs). BEVs and MEVs have many similarities in common, such as their hollow nanostructure comprised of lipid bilayer membrane, stability in physiological environments, and their intrinsic targeting abilities attributed to their membrane proteome. These advantages have been exploited by researchers for the development of extracellular vesicle-based delivery platforms for biomedical applications. Notably, BEVs have several notable characteristics when compared to MEVs, suggesting BEV’s potential as promising nanopharmaceuticals, such as stability in the living system, penetrative capabilities, as well as ease of engineering and industrial production. One of the advantages of BEVs is their high yield and cost-effective production. As derivatives of bacteria, BEV production benefits from the advantages of industrial-scale bacteria cultivation. The short doubling time of bacteria allows for high cell density liquid cultures, which rely on cheap and readily available liquid medium, ensuring cost-effectivity [241]. Furthermore, hyper-vesiculating mutants of *H. pylori* and *E coli* have been developed to further increase the yields of BEVs [242, 243]. On the other hand, the production of MEVs often suffers from long culturing periods and low yields of exosomes [244]. Potentially, BEVs can serve as economical drug delivery agents with wide applicability across various therapeutic applications.

The lipid membranes of BEVs are also highly stable and resistant to a wide variety of enzymatic activities such as proteases and nucleases [245, 246]. This characteristic makes BEVs suitable vehicles for biomolecular-based cargoes that are sensitive to temperatures and enzymatic activities, such as proteins, enzymes, or genetic materials [245, 247–249]. By storing such sensitive biomolecules in BEVs, they can be delivered intact to their target site without degradation or loss of function, addressing a common issue that limits clinical implementation [250]. Additionally, BEVs can function as stabilizing “packages” allowing long-term storage of biomolecules at elevated temperatures. For example, when phosphotriesterase (PTE) was packaged into *E. Coli* BEVs survived storage at 37 °C for 14 days and multiple freeze–thaw cycles, preserving higher enzymatic activity compared to free PTE [245]. This ability suggests that heat-sensitive cargoes can be transported in BEVs without the need for expensive logistical processes (such as cryostorage) for extended periods [251], unlike SARS-COV -mRNA vaccines encapsulated in liposomes [252]. The effects of storage temperature on the quality of MEVs and their interior cargo remains inconclusive. Generally, MEVs may be less robust and unstable than BEVs, as the storage of MEVs requires specialized buffers and storage temperature of 4–20 °C for optimal activity [253, 254]. The remarkable ability of BEVs to stabilize and protect cargoes from physiological and physical environments makes them ideal carriers for drug delivery.

BEVs are excellent nanocarriers, as they have been reported to be able to penetrate the biological tissue barrier, such as the epithelial cell layer [252], target skeletal structures [110], and even bypass the blood–brain barrier (BBB) [255, 256]. This outstanding capability of BBB penetration addresses a major limitation in the progression of many promising neurological drug candidates targeting the Central Nervous System [257]. Notably, BEVs also exhibit outstanding targeting capabilities which are attributed to their membrane composition and intrinsic protein expression. The similarities in the membrane composition allow BEVs to have the affinity to parental bacteria whole cells while certain antigens can allow them to target bacteria from other species or strains [8, 258, 259]. Synergizing with their penetrative ability across tissue and cellular barriers, BEVs can realize precise biomedical strategies for bacterial infections. Furthermore, BEVs play a key role in the interaction between the host system and bacteria, by internalizing into mammalian cells through various pathways such as endocytosis, membrane fusion, and receptor-mediated signaling [35], potentiating the immune system activation for therapy. However, due to the difference in origin and membrane composition, MEVs lack bacterial cell targeting capabilities and are limited to only host or mammalian cells, restricting their strategic application in bacterial-induced diseases. Therefore, benefiting from their unobstructed bypass through various barriers, affinity to homotypic bacterial cells, and mediating the interaction between host cells and bacteria, BEVs can be employed as carriers to potentially revive previously unsuccessful therapeutics plagued with distribution, permeation, and stability issues in vivo [260].

Notably, owing to the mature genetic manipulation of bacteria, the expression of surface proteins, and functional sites on their membrane, the exterior of BEVs can be easily engineered for cargo loading or the regulation of their interactions with target cells [261, 262]. BEVs can be modified by cultivation in modified mediums, or even hybridization with other forms of nanoparticles and nanovesicles as exemplified in this review. This allows the development of facile BEV-based nanopharmaceuticals with diverse biomedical applications. Furthermore, isolated BEVs can also be further decorated with functional ligands via protein–ligand interactions [263], protein–protein interaction [264], or potentially bioconjugate reactions. Overall, these engineering strategies result in BEVs which are highly precise delivery platforms with improved therapeutic efficiencies to combat extensive biomedical challenges. As MEVs also originate from cells and have a similar membrane structure as BEVs, they can be similarly modified as BEVS, but the genetic modification of mammalian cells is generally more difficult as compared to bacteria, greatly limiting their scope of application.

Importantly, the advantage of BEVs is their immunogenic properties and ability to engage with the immune system. Other than simply serving as nanocarriers for drug delivery, BEVs can also serve as functional nanoagents that can stimulate immune responses which may augment the therapeutic effects of their cargo [140]. Furthermore, the immunogenicity of BEVs allows them to be utilized as vaccines to stimulate long-term immune response or act as adjuvants in combinational vaccines against cancers, and bacterial and viral infections [129, 265]. MEVs which are derived from mammalian cells have limited immunogenicity and lack exogenous antigen expression, impeding their immune response for therapy. MEVs have also been capitalized to develop vaccine platforms, but they have a narrower scope when compared to BEVs as they are mainly focused on antitumoral applications [266]. The unique immunogenicity of BEVs qualifies them to be a competitive platform for the development of functional nanopharmaceuticals.

## Conclusion and further perspective

In this review, we comprehensively summarize the development of BEV-based nanopharmaceuticals to facilitate disparate biomedical applications in recent years. Amongst the unique advantages and functions of BEVs, we demonstrated the tremendous potential of applying these naturally occurring nanovesicles to establish a myriad of innovative therapeutic strategies for new-generation pharmaceuticals evolution, especially in the versatile bioactive cargo delivery and powerful vaccination approaches.

Taking the unique merits of nanovesicles, the structural stability, ease of cargo loading, promising penetration across physiological barriers, and specific targeting capabilities endow the BEVs application to be beneficial in delivery for a wide range of therapeutics. Particularly, the naturally occurring membrane structure of BEVs enables targeting delivery of therapeutic genetic tools (e.g., siRNA, DNA, CRISPR-Cas9, etc.) in the physiological, preserving stability and bioactivity, thus enhancing gene therapy. Leveraging the ease of genetic modification in parent bacteria, protein cargo, such as enzymes and antigens, can also be directly expressed within BEVs, optimizing loading efficiency compared to conventional delivery platforms. Furthermore, advancements in nanotechnology and material science have witnessed the integration of functional materials with BEV delivery platforms to achieve combinational therapy, thereby further improving the synergistic therapeutic efficacy.

However, several considerations regarding the optimization of BEV-based delivery platforms are still necessary to be taken care to propel the BEV nanopharmaceuticals into clinical translation. The current isolation of BEVs relies heavily on ultracentrifugation, which raises the concern of a time-consuming and energy-intensive nature [21]. The established technique cannot fully separate the BEVs from the lysis of parent bacteria, resulting in low purity of nanovesicles and making BEVs identification challenging [267]. Several affinity-based purification techniques (e.g. magnetic bead-mediated adsorption, antibody-based selection, etc.) [268, 269] resulting in high yield and purity of mammalian extracellular vesicles have been proposed and can be potentially translated for BEV isolation to increase their yield and achieve higher purities. Besides, the larger-scale production of BEVs from living bacteria may lead to inconsistencies in size and overall composition from batch to batch. Enhancements in standard harvesting procedures for BEVs and the culturing of parent bacteria are imperative.

Notably, by thanking the abundant display of native PAMPs and immunostimulatory antigens, BEVs exhibit unique immunogenicity making them promising vaccine platforms, while the self-adjuvating properties of BEVs stimulate host immune responses. BEV-based nanovaccines have demonstrated efficacy in preventing bacterial and viral infections, as well as combating tumors, highlighting their potential in addressing a wide range of diseases. Despite numerous promising achievements, the clinical practice of the BEV-based vaccination method remains controversial. It's crucial to recognize that PAMPs play a significant and intrinsic role in the pathogenicity of bacteria. Thus, the balance between biosafety and immunostimulatory of BEVs-based vaccines should be carefully considered. The inadvertent introduction of virulent factors (e.g. LPS and virulent protein) in extracted BEVs can lead to excessive immune stimulation, inflammatory responses, reactogenicity, or other adverse effects [259, 270, 271]. Moving forward, it is imperative to elucidate the pathogenicity of harmful components inherited by BEVs. Further investigation is necessary to develop improved methods for isolating and purifying BEVs, ensuring the removal of detrimental bacterial components that may pose a threat to the host before implementation, thereby minimizing adverse effects [272, 273]. This is especially critical when administering BEVs to immunocompromised individuals [274].

In conclusion, we believe that bacterial extracellular vesicles have emerged as a new era of innovative nanopharmaceuticals attributed to their outstanding advantages and attractive functions. Continued studies will undoubtedly explore the vast potential of BEVs as an indispensable and influential tool to boost biomedical applications, paving the way for their clinical translation and revolution of nanomedicine.

## Data Availability

The review is based on the published data and sources of data upon which conclusions have been drawn can be found in the reference list.

## Abbreviations

BEV: Bacterial extracellular vesicle
LPS: Lipopolysaccharide
CME: Clathrin-mediated endocytosis
TLR: Toll-like receptors
PAMP: Pathogen-associated molecular patterns
PRR: Pathogen recognition receptors
IL: Interleukin
TNF: Tumour necrosis factor
AMP: Antimicrobial peptides
hBD: Human-β-defensin
BBB: Blood–brain barrier
Rif: Rifampicin
OMP: Outer membrane protein
Ig: Immunoglobulin
NSCLC: Non-small-cell lung cancer
DOX: Doxorubicin
APEC: Avian pathogenic *Escherichia coli*
CRISPR: Clustered regulatory interspaced short tandem repeats
OIMV: Outer-inner membrane vesicles
EOMV: Explosive outer membrane vesicles
CMV: Membrane vesicles
CT: Cholera toxin
PDT: Photodynamic
ICG: Indocyanine green
